# Impact of neuraminidase inhibitors on influenza A(H1N1)pdm09‐related pneumonia: an individual participant data meta‐analysis

**DOI:** 10.1111/irv.12363

**Published:** 2016-02-01

**Authors:** Stella G. Muthuri, Sudhir Venkatesan, Puja R. Myles, Jo Leonardi‐Bee, Wei Shen Lim, Abdullah Al Mamun, Ashish P. Anovadiya, Wildo N. Araújo, Eduardo Azziz‐Baumgartner, Clarisa Báez, Carlos Bantar, Mazen M. Barhoush, Matteo Bassetti, Bojana Beovic, Roland Bingisser, Isabelle Bonmarin, Victor H. Borja‐Aburto, Bin Cao, Jordi Carratala, María R. Cuezzo, Justin T. Denholm, Samuel R. Dominguez, Pericles A. D. Duarte, Gal Dubnov‐Raz, Marcela Echavarria, Sergio Fanella, James Fraser, Zhancheng Gao, Patrick Gérardin, Maddalena Giannella, Sophie Gubbels, Jethro Herberg, Anjarath L. Higuera Iglesias, Peter H. Hoeger, Matthias Hoffmann, Xiaoyun Hu, Quazi T. Islam, Mirela F. Jiménez, Amr Kandeel, Gerben Keijzers, Hossein Khalili, Gulam Khandaker, Marian Knight, Gabriela Kusznierz, Ilija Kuzman, Arthur M. C. Kwan, Idriss Lahlou Amine, Eduard Langenegger, Kamran B. Lankarani, Yee‐Sin Leo, Rita Linko, Pei Liu, Faris Madanat, Toshie Manabe, Elga Mayo‐Montero, Allison McGeer, Ziad A. Memish, Gokhan Metan, Dragan Mikić, Kristin G. I. Mohn, Ahmadreza Moradi, Pagbajabyn Nymadawa, Bulent Ozbay, Mehpare Ozkan, Dhruv Parekh, Mical Paul, Wolfgang Poeppl, Fernando P. Polack, Barbara A. Rath, Alejandro H. Rodríguez, Marilda M. Siqueira, Joanna Skręt‐Magierło, Ewa Talarek, Julian W. Tang, Antoni Torres, Selda H. Törün, Dat Tran, Timothy M. Uyeki, Annelies van Zwol, Wendy Vaudry, Daiva Velyvyte, Tjasa Vidmar, Paul Zarogoulidis, PRIDE Consortium Investigators, Jonathan S. Nguyen‐Van‐Tam, Maria de Lourdes Aguiar‐Oliveira, Tarig SA Al Khuwaitir, Malakita Al Masri, Robed Amin, Elena Ballester‐Orcal, Jing Bao, Ariful Basher, Edgar Bautista, Barbara Bertisch, Julie Bettinger, Robert Booy, Emilio Bouza, Ilkay Bozkurt, Heinz Burgmann, Elvira Čeljuska‐Tošev, Kenny KC Chan, Yusheng Chen, Tserendorj Chinbayar, Catia Cilloniz, Rebecca J Cox, Elena B Sarrouf, Wei Cui, Simin Dashti‐Khavidaki, Bin Du, Hicham El Rhaffouli, Hernan Escobar, Agnieszka Florek‐Michalska, John Gerrard, Stuart Gormley, Sandra Götberg, Behnam Honarvar, Jianming Hu, Christoph Kemen, Evelyn SC Koay, Miroslav Kojic, Koichiro Kudo, Win M Kyaw, Leonard Leibovici, Xiao‐li Li, Hongru Li, Romina Libster, Tze P Loh, Deborough Macbeth, Efstratios Maltezos, Débora N Marcone, Magdalena Marczynska, Fabiane P Mastalir, Auksė Mickiene, Mohsen Moghadami, Lilian Moriconi, Maria E Oliva, Blaž Pečavar, Philippe G Poliquin, Mahmudur Rahman, Alberto Rascon‐Pacheco, Samir Refaey, Brunhilde Schweiger, Anna C Seale, Bunyamin Sertogullarindan, Fang G Smith, Ayper Somer, Thiago ML Souza, Frank Stephan, Payam Tabarsi, CB Tripathi, Diego Viasus, Qin Yu, Wei Zhang, Wei Zuo

**Affiliations:** ^1^ Division of Epidemiology and Public HealthUniversity of NottinghamNottinghamUK; ^2^ Respiratory MedicineNottingham University Hospitals NHS TrustNottinghamUK; ^3^ International Centre for Diarrhoeal DiseasesResearch Bangladesh (ICDDRB)DhakaBangladesh; ^4^ Department of PharmacologyGovernment Medical College and Sir Takhtsinhji General HospitalBhavnagarGujaratIndia; ^5^ University of BrasíliaBrasíliaDFBrazil; ^6^ Centers for Disease Control and PreventionAtlantaGAUSA; ^7^ Ministerio de Salud de la Provincia de Buenos AiresBuenos AiresArgentina; ^8^ Department of Infection ControlHospital San Martín de ParanáEntre RíosArgentina; ^9^ Department of MedicineKing Saud Medical CityRiyadhSaudi Arabia; ^10^ Santa Maria Misericordia HospitalUdineItaly; ^11^ Department of Infectious DiseasesUniversity Medical CentreLjubljanaSlovenia; ^12^ Department of Emergency MedicineUniversity Hospital BaselBaselSwitzerland; ^13^ Institut de Veille SanitaireSaint‐MauriceFrance; ^14^ Instituto Mexicano del Seguro Social (IMSS)Mexico CityMexico; ^15^ Beijing Chao‐Yang HospitalCapital Medical UniversityBeijingChina; ^16^ Department of Infectious DiseasesHospital Universitari de BellvitgeBellvitge Institute for Biomedical ResearchL'Hospitalet de LlobregatRed Española de Investigación en Patología InfecciosaUniversity of BarcelonaBarcelonaSpain; ^17^ Ministerio de Salud de TucumánTucumánArgentina; ^18^ Victorian Infectious Diseases Service and Department of Microbiology and ImmunologyPeter Doherty Institute for Infection and ImmunityParkvilleVic.Australia; ^19^ Department of Pediatric Infectious DiseasesChildren's Hospital ColoradoUniversity of Colorado School of MedicineAuroraCOUSA; ^20^ Universidade Estadual do Oeste do ParanáUNIOESTECascavelPRBrazil; ^21^ The Edmond and Lily Safra Children's HospitalSheba Medical CenterTel‐HashomerIsrael; ^22^ Clinical Virology LaboratoryCEMIC University HospitalBuenos AiresArgentina; ^23^ Section of Pediatric Infectious DiseasesUniversity of ManitobaWinnipegMBCanada; ^24^ Paediatric Intensive Care UnitBristol Children's HospitalBristolUK; ^25^ Department of Respiratory & Critical Care MedicinePeking University People's HospitalBeijingChina; ^26^ NICU/PICUPFMECHU Saint PierreSaint PierreLa RéunionFrance; ^27^ CIC 1410 (CHU/Inserm/University of La Réunion/URML‐OI)CHU Saint PierreSaint PierreLa RéunionFrance; ^28^ UMR PIMIT (CHU/Inserm/University of La Réunion/IRD/CNRS)CYROISaint Denis – Reunion IslandSaint DenisFrance; ^29^ NICU/PICU CHU of La RéunionGroupe Hospitalier Sud RéunionSaint PierreLa RéunionFrance; ^30^ Department of Clinical Microbiology and Infectious DiseasesHospital General Universitario Gregorio MarañónMadridSpain; ^31^ Department of Infectious Disease EpidemiologySector for National Health Documentation and ResearchStatens Serum InstitutCopenhagenDenmark; ^32^ Section of PaediatricsDivision of Infectious DiseaseImperial CollegeLondonUK; ^33^ Epidemiology Research UnitInstituto Nacional de Enfermedades RespiratoriasIsmael Cosío VillegasMexico CityMexico; ^34^ Cath. Children's Hospital WilhelmstiftHamburgGermany; ^35^ Division of Infectious Diseases and Hospital EpidemiologyKantonsspital St. GallenSt. GallenSwitzerland; ^36^ Peking Union Medical College HospitalBeijingChina; ^37^ Dhaka Medical College HospitalDhakaBangladesh; ^38^ Departamento de Ginecologia e Obstetrícia – UFCSPAPreceptora da Residência Médica do Hospital FêminaPorto AlegreBrazil; ^39^ Ministry of Health in EgyptCairoEgypt; ^40^ Gold Coast HospitalGold CoastQldAustralia; ^41^ Department of Clinical PharmacyFaculty of PharmacyTehran University of Medical SciencesTehranIran; ^42^ National Centre for Immunisation Research and Surveillance (NCIRS)The Children's Hospital at WestmeadUniversity of SydneySydneyNSWAustralia; ^43^ National Perinatal Epidemiology UnitNuffield Department of Population HealthUniversity of OxfordOxfordUK; ^44^ National Institute of Respiratory Diseases ‘Emilio Coni’ ANLIS “C. Malbran”Santa FeArgentina; ^45^ School of MedicineUniversity Hospital for Infectious DiseasesUniversity of ZagrebZagrebCroatia; ^46^ Department of Intensive CarePamela Youde Nethersole Eastern HospitalChai WanHong Kong; ^47^ Faculty of Medicine and PharmacyMohammed V Military Teaching HospitalBiosafety Level 3 and Research LaboratoryUniversity Mohammed V‐SouissiRabatMorocco; ^48^ Department of Obstetrics and GynaecologyStellenbosch University and TygerbergStellenboschSouth Africa; ^49^ Health Policy Research CenterShiraz University of Medical SciencesShirazIran; ^50^ Department of Infectious DiseasesTan Tock Seng HospitalSingaporeSingapore; ^51^ Helsinki University HospitalHelsinkiFinland; ^52^ Department of Infectious DiseasesThe First Affiliated HospitalChina Medical UniversityShenyangChina; ^53^ Department of PediatricsKing Hussein Cancer CenterAmmanJordan; ^54^ Graduate School of Comprehensive Human SciencesUniversity of TsukubaTsukubaIbarakiJapan; ^55^ Instituto de Medicina Preventiva de la DefensaCapitan Medico Ramon y Cajal (IMPDEF)Ministerio de DefensaMadridSpain; ^56^ Toronto Invasive Bacterial Diseases NetworkUniversity of TorontoTorontoONCanada; ^57^ Ministry of HealthRiyadhSaudi Arabia; ^58^ College of MedicineAlfaisal UniversityRiyadhSaudi Arabia; ^59^ Department of Infectious Diseases and Clinical MicrobiologyErciyes University Faculty of MedicineKayseriTurkey; ^60^ Military Medical AcademyClinic for Infectious and Tropical DiseasesBelgradeSerbia; ^61^ Section for Infectious DiseasesMedical Department, and Department of Research and DevelopmentHaukeland University HospitalBergenNorway; ^62^ Department of Clinical ScienceThe Influenza CentreUniversity of BergenBergenNorway; ^63^ The Division of Ocular ImmunologyDepartment of OphthalmologyJohns Hopkins University School of MedicineBaltimoreMDUSA; ^64^ National Research Institute for Tuberculosis and Lung DiseaseMassih Daneshvari HospitalShahid Beheshti University of Medical SciencesTehranIran; ^65^ National Influenza CenterNational Center of Communicable DiseasesMinistry of HealthUlaanbaatarMongolia; ^66^ Department of Pulmonary and Critical CareYuzuncu Yil University Medical FacultyVanTurkey; ^67^ Clinic of Pediatric NeurologyDr. Sami Ulus Research and Training Hospital of Women's and Children's Health and DiseasesAnkaraTurkey; ^68^ Critical Care and Pain PerioperativeCritical Care and Trauma Trials GroupSchool of Clinical and Experimental MedicineUniversity of BirminghamBirminghamUK; ^69^ Division of Infectious DiseasesRambam Health Care CampusHaifaIsrael; ^70^ Medical University of ViennaViennaAustria; ^71^ Department of PediatricsVanderbilt Vaccine CenterVanderbilt UniversityNashvilleTNUSA; ^72^ Fundacion INFANTBuenos AiresArgentina; ^73^ Division of Pneumonology‐ImmunologyDepartment of PediatricsCharité University Medical CenterBerlinGermany; ^74^ Critical Care DepartmentHospital Joan XXIIIIISPVURVCIBERESTarragonaSpain; ^75^ Laboratory of Respiratory VirusesOswaldo Cruz Institute/FiocruzRio de JaneiroBrazil; ^76^ Uniwersytet RzeszowskiRzeszówPoland; ^77^ Department of Children's Infectious DiseasesMedical University of WarsawWarsawPoland; ^78^ Division of Microbiology/Molecular Diagnostic CentreDepartment of Laboratory MedicineNational University HospitalSingaporeSingapore; ^79^ Alberta Provincial Laboratory for Public HealthUniversity of Alberta HospitalEdmontonCanada; ^80^ Department of Medical Microbiology and ImmunologyUniversity of AlbertaEdmontonABCanada; ^81^ Hospital ClinicUniversity of BarcelonaIDIBAPSCIBERESBarcelonaSpain; ^82^ Department of Pediatric Infectious DiseasesIstanbul Medical FacultyIstanbulTurkey; ^83^ Division of Infectious DiseasesDepartment of PaediatricsThe Hospital for Sick ChildrenUniversity of TorontoCanada; ^84^ Influenza DivisionNational Center for Immunization and Respiratory DiseasesCenters for Disease Control and PreventionAtlantaGAUSA; ^85^ Department of Pediatric Intensive CareVU University Medical CenterAmsterdamThe Netherlands; ^86^ Division of Infectious DiseasesDepartment of PediatricsStollery Children's HospitalUniversity of AlbertaEdmontonABCanada; ^87^ Lithuanian University of Health SciencesKaunasLithuania; ^88^ General HospitalSlovenj GradecSlovenia; ^89^ Unit of Infectious DiseasesUniversity General Hospital of AlexandroupolisDemocritus University ThraceDraganaGreece

**Keywords:** Hospitalisation, individual participant data meta‐analyses, influenza‐related pneumonia, neuraminidase inhibitors

## Abstract

**Background:**

The impact of neuraminidase inhibitors (NAIs) on influenza‐related pneumonia (IRP) is not established. Our objective was to investigate the association between NAI treatment and IRP incidence and outcomes in patients hospitalised with A(H1N1)pdm09 virus infection.

**Methods:**

A worldwide meta‐analysis of individual participant data from 20 634 hospitalised patients with laboratory‐confirmed A(H1N1)pdm09 (*n* = 20 021) or clinically diagnosed (*n* = 613) ‘pandemic influenza’. The primary outcome was radiologically confirmed IRP. Odds ratios (OR) were estimated using generalised linear mixed modelling, adjusting for NAI treatment propensity, antibiotics and corticosteroids.

**Results:**

Of 20 634 included participants, 5978 (29·0%) had IRP; conversely, 3349 (16·2%) had confirmed the absence of radiographic pneumonia (the comparator). Early NAI treatment (within 2 days of symptom onset) versus no NAI was not significantly associated with IRP [adj. OR 0·83 (95% CI 0·64–1·06; *P* = 0·136)]. Among the 5978 patients with IRP, early NAI treatment versus none did not impact on mortality [adj. OR = 0·72 (0·44–1·17; *P* = 0·180)] or likelihood of requiring ventilatory support [adj. OR = 1·17 (0·71–1·92; *P* = 0·537)], but early treatment versus later significantly reduced mortality [adj. OR = 0·70 (0·55–0·88; *P* = 0·003)] and likelihood of requiring ventilatory support [adj. OR = 0·68 (0·54–0·85; *P* = 0·001)].

**Conclusions:**

Early NAI treatment of patients hospitalised with A(H1N1)pdm09 virus infection versus no treatment did not reduce the likelihood of IRP. However, in patients who developed IRP, early NAI treatment versus later reduced the likelihood of mortality and needing ventilatory support.

## Introduction

Influenza‐related pneumonia (IRP) was a common and severe complication during the 2009–2010 influenza pandemic.^1^, ^2^, ^3^, ^4^, ^5^ Neuraminidase inhibitors (NAIs), primarily oseltamivir and zanamivir, were widely recommended for patients with suspected or confirmed influenza A(H1N1)pdm09 virus infection.^6^, ^7^ However, prior to the 2009–2010 pandemic, evidence of their effectiveness in seasonal influenza, while strong for modest symptom alleviation, was less robust for reductions in pneumonia incidence or improvements in pneumonia outcome.^8^, ^9^, ^10^ The findings from meta‐analyses have been inconsistent. One study based on observational data from 150 660 patients with mainly seasonal influenza suggested no statistically significant reduced likelihood of pneumonia.^9^ Another used clinical trials data from 4452 community adult patients with uncomplicated seasonal influenza and concluded that oseltamivir significantly reduced ‘self‐reported, investigator‐mediated, unverified pneumonia’ by 45%, compared with placebo, but data on radiologically confirmed pneumonia were not available.^11^

A recent individual participant data (IPD) analysis of clinical trial data investigating the efficacy of oseltamivir when compared to placebo in patients with seasonal influenza reported a reduction in risk of pneumonia by 60%.^12^ Individual observational studies during the 2009–2010 pandemic suggest a possible benefit of NAIs in reducing pneumonia incidence, but are limited by small sample sizes.^13^, ^14^, ^15^, ^16^ A meta‐analysis of 2009–2010 pandemic data from patients hospitalised with influenza A(H1N1)pdm09 virus infection reported that early treatment with NAIs reduced the likelihood of IRP compared to late treatment by 65%.^17^ But this work encountered high degrees of heterogeneity and inconsistent or incomplete adjustment for potential confounders.

We present a global meta‐analysis based on IPD, controlling for potential confounders and treatment propensity. We investigate the association between NAI treatment and radiologically confirmed IRP in patients hospitalised with A(H1N1)pdm09 virus infection, and outcomes including admission to intensive care units (ICUs), ventilatory support, acute respiratory distress syndrome (ARDS) and mortality in patients with IRP.

Some of these results have been previously reported in the form of an abstract.^18^

## Methodology

### The PRIDE research consortium

**Figure 1:**
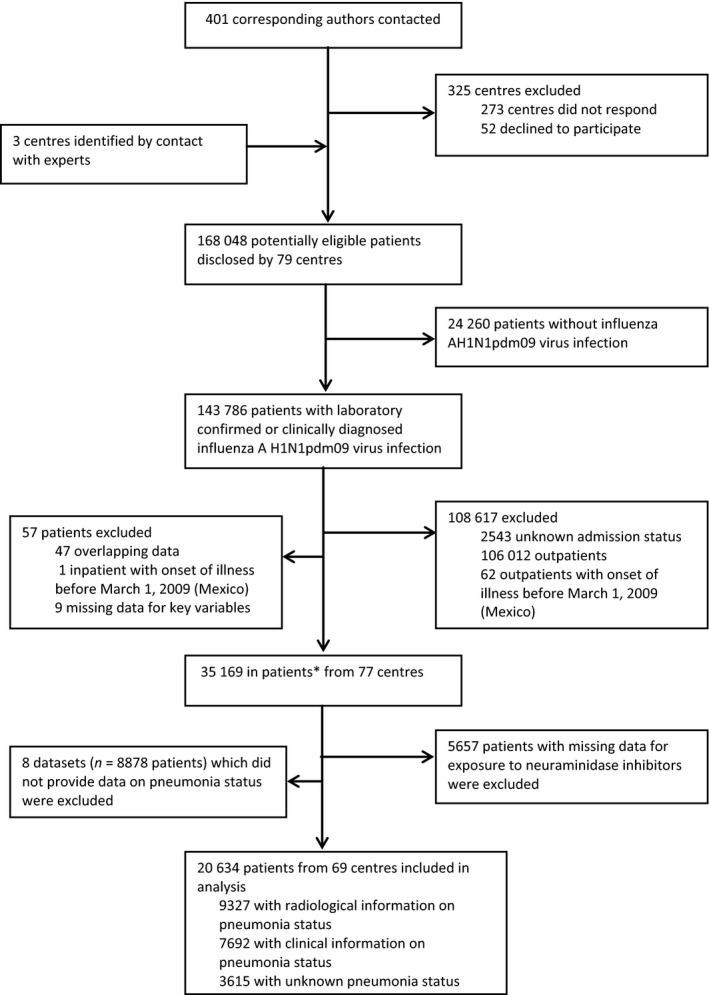
Study flow diagram. *Two hundred and sixty patients added since publication of Muthuri *et al*.^17^ following clarification of inpatient status from data collaborator.

Details of the Post‐pandemic Review of anti‐Influenza Drug Effectiveness (PRIDE) study have been published previously.^19^ Briefly, participating research centres were identified during the conduct of a systematic review of published studies on the same topic.^17^ Additional centres were recruited through this network of global collaborators, publicity at conferences and by word of mouth. Centres that fulfilled the minimum data set requirements (Table S2) were eligible for inclusion in the consortium. In total, 79 research groups from 38 countries and six World Health Organization (WHO) regions contributed data on 143 786 patients with laboratory‐ or clinically diagnosed influenza A(H1N1)pdm09 virus infection (Figure 1). No data were provided or funded for collection by pharmaceutical companies. The protocol was registered with the PROSPERO register of systematic reviews, number CRD42011001273.^20^

### Data standardisation, exposure and outcome variables

Data were standardised using a common data dictionary^19^ before pooling for analysis. For this analysis, the primary outcome was IRP defined as laboratory‐confirmed or clinically diagnosed influenza A(H1N1)pdm09 virus infection plus pneumonia confirmed by chest radiography, occurring at any time after the onset of influenza‐like illness. For radiographic evidence of pneumonia, we accepted:

1. A formal chest radiograph or computerised tomograph report documenting ‘pneumonia’.
2. Data sets reporting pneumonia and chest radiograph as discrete variables, in which both items were marked positive or ‘yes’.
3. Formal chest radiograph reports of one or more abnormalities consistent with pneumonia: pulmonary infiltrates, lobar consolidation, homogeneous segmental consolidation with or without cavitation, diffuse bilateral interstitial and/or interstitial–alveolar (mixed) infiltrates, segmental consolidation, lobar consolidation, rounded pneumonia, bronchopneumonia, interstitial pneumonia, pneumatoceles, acute pulmonary infiltrates, as previously validated by Bewick *et al*. and Franquet,^21^, ^22^ unless a formal radiograph report also stated ‘no pneumonia’.
4. Chest radiograph report not provided, but specific mention in the clinical case notes that a radiograph had been formally reported as showing pneumonia.

The absence of IRP (‘no IRP’) was defined as laboratory‐confirmed or clinically diagnosed influenza A(H1N1)pdm09 infection plus a radiographic report that did not identify abnormalities consistent with pneumonia, or which stated that pneumonia was ‘not present’ (irrespective of any specific features reported).

Comparative exposure to NAI treatment was defined as follows: early NAI treatment (≤2 days after symptom onset) versus no NAI treatment; early NAI treatment versus later NAI treatment (treatment commenced >2 days after symptom onset); later NAI treatment versus no NAI treatment; and NAI treatment (irrespective of timing) versus no NAI treatment.

### Propensity scoring

Propensity scores for the likelihood of NAI treatment were calculated for each patient within individual data sets using multivariable logistic regression for each of the three NAI exposure measures, using covariates as described by Muthuri *et al*.^19^ (Table S3). Subsequently, propensity scores were categorised into quintiles for each individual data set.

### Statistical analysis

To investigate the association between the use of NAI treatment and IRP, we compared patients with IRP against those with no IRP. We used generalised linear mixed modelling to conduct separate analyses for each NAI exposure comparison using the xtmelogit command in stata (version 13; StataCorp LP, College Station, TX, USA). Individual studies were included in the model as a random intercept in order to account for differences in baseline outcome. Adjustment was performed for propensity of NAI treatment, antibiotics administered during hospitalisation and corticosteroids administered during hospitalisation. Missing data in the covariates were included as a separate dummy category to allow for comparisons across the crude and adjusted analyses. We excluded data sets in which all patients (*n* = 1352 from 14 data sets) were diagnosed with IRP. Stratified analyses were conducted for adults (≥16 years), children (<16 years; including <5‐ and 5‐ to 15‐year subgroups), pregnant women, laboratory‐confirmed A(H1N1)pdm09 cases and patients admitted to critical care units. We did not include patients with unknown pneumonia status (*n* = 3615 across 21 data sets) in this analysis.

In the subgroup of patients with IRP, we further examined the effect of NAI treatment on secondary clinical outcomes: admission to ICUs, ventilatory support, ARDS and mortality. At this juncture, we re‐included the 14 data sets in which all patients were diagnosed with IRP.

### Sensitivity analysis

In some clinical settings, chest radiography is not routinely performed for hospitalised patients with influenza unless a pulmonary complication is also suspected; therefore, reliance on radiographic abnormalities is likely to give a conservative estimate of pneumonia incidence. Accordingly, we also performed a sensitivity analysis, which considered a diagnosis of ‘any pneumonia’ by combining IRP with physician‐diagnosed pneumonia (PDP), the latter defined as laboratory‐confirmed or clinically diagnosed influenza A(H1N1)pdm09 plus a physician diagnosis of pneumonia, but where no chest radiograph report was available. For this analysis, patients categorised as ‘no pneumonia’ had laboratory‐confirmed or clinically diagnosed influenza A(H1N1)pdm09 with no evidence of IRP on chest radiography; unknown pneumonia status; or, in the absence of a chest radiograph report, no documented clinical record of PDP, recognising that clinicians record positive findings in the case record, but not all negative findings.

Results are presented as unadjusted and adjusted odds ratios (OR) with 95% confidence intervals (95% CI), and two‐sided *P*‐values < 0·05 were considered statistically significant. Statistical analyses were conducted using stata (version 13).

## Results

Overall, data were obtained on 35 169 individuals hospitalised with A(H1N1)pdm09 virus infection (Figure 1). Of these, 29 512 (84%) patients were admitted from January 2009 through March 2011 (Figure S1) with information available on NAI treatment. A further eight data sets comprising 8878 hospitalised patients that did not provide data on pneumonia status were excluded from the analysis (Figure 1; Table S4).

Of the 20 634 patients included, 9327 (45%) had a positive or negative diagnosis of IRP confirmed by chest radiography, while 7692 (37%) did not have chest radiography, but had a positive or negative diagnosis of PDP documented. The remaining 3615 (18%) hospitalised patients had neither radiological nor clinical documentation of pneumonia status; they were included in the sensitivity analysis (only) as having ‘no pneumonia’. The characteristics of hospitalised patients with and without pneumonia included in the pooled data set are shown in Table 1. Baseline characteristics of each constituent data set included in the analysis are presented in Table S5.

**Table 1:** Characteristics of pooled data set of 20 634 patients admitted to hospital with influenza A(H1N1)pdm09 virus infection with and without pneumonia

Characteristics	Radiologically diagnosed pneumonia status		Radiologically or PDP status	
	IRP	No IRP	Any pneumonia^a^	No pneumonia^b^
Number of patients^c^	5978 (100·0)	3349 (100·0)	7054 (100·0)	13 580 (100·0)
Number of male cases	3266 (54·6)	1879 (56·0)	3811 (54·0)	6645 (48·9)
Age: median (IQR) in years	36 (17–52)	26 (14–46)	35 (14–51)	22 (8–38)
Adults (≥16 years)	4560 (76·3)	2436 (72·7)	5208 (73·8)	8482 (62·5)
Children (<16 years)	1411 (23·6)	912 (27·2)	1821 (25·8)	4966 (36·6)
Obese^d^	952 (15·9)	229 (6·8)	1072 (15·2)	744 (5·5)
Smoking	914 (15·3)	481 (14·4)	958 (13·6)	867 (6·4)
Pregnant women^e^	219 (13·1)	150 (16·0)	279/1967 (14·2)	1153/4397 (26·2)
WHO regions				
African region	28 (0·5)	1 (0·03)	31 (0·4)	10 (0·1)
Region of the Americas	2314 (38·7)	550 (16·4)	2703 (38·3)	4948 (36·4)
Eastern Mediterranean region	178 (3·0)	206 (6·2)	549 (7·8)	3086 (22·7)
European region	2635 (44·1)	2032 (60·7)	2932 (41·6)	4080 (30·0)
South‐East Asia region	45 (0·8)	86 (2·6)	45 (0·6)	157 (1·2)
Western Pacific region	778 (13·0)	474 (14·2)	794 (11·3)	1299 (9·6)
A(H1N1)pdm09 diagnosis				
Laboratory confirmed	5755 (96·3)	3146 (93·9)	6827 (96·8)	13 194 (97·2)
Clinically diagnosed	223 (3·7)	203 (6·1)	227 (3·2)	386 (2·8)
Comorbidities^f^				
Any comorbidity	3021 (50·5)	1795 (53·6)	3531 (50·1)	5449 (40·1)
Asthma	856 (14·3)	777 (22·7)	968 (13·7)	1430 (10·5)
COPD	432 (7·2)	249 (7·4)	454 (6·4)	345 (2·5)
Other chronic lung disease	492 (8·2)	525 (15·7)	648 (9·2)	1668 (12·3)
Heart disease	650 (10·9)	341 (10·2)	713 (10·1)	786 (5·8)
Renal disease	278 (4·7)	113 (3·4)	328 (4·7)	349 (2·6)
Liver disease	122 (2·0)	73 (2·2)	127 (1·8)	121 (0·9)
Cerebrovascular disease	121 (2·0)	122 (3·6)	133 (1·9)	170 (1·3)
Neurological disease	436 (7·3)	237 (7·1)	492 (7·0)	508 (3·7)
Diabetes	634 (10·6)	280 (8·4)	725 (10·3)	690 (5·1)
Immunosuppression	525 (8·8)	242 (7·2)	610 (8·7)	852 (6·3)
H1N1pdm09 vaccination^g^	121/2917 (4·2)	48/1701 (2·8)	163/3738 (4·4)	176/6237 (2·8)
Time from symptom onset to hospital admission, days, median (IQR)	4 (2–6)	2 (1–4)	3 (2–6)	2 (1–4)
Time from symptom onset to antiviral treatment, days, median (IQR)	4 (2–7)	2 (1–4)	4 (2–7)	2 (1–4)
Antiviral agents used				
No NAI treatment	582 (9·7)	540 (16·1)	724 (10·3)	4336 (31·9)
Any NAI	5396 (90·3)	2809 (83·9)	6330 (89·7)	9244 (68·1)
Oral oseltamivir^h^	5356 (99·3)	2782 (99·0)	6263 (98·9)	9068 (98·1)
Intravenous/inhaled zanamivir^h^	134 (2·5)	40 (1·4)	155 (2·5)	158 (1·7)
Intravenous peramivir^h^	42 (0·8)	5 (0·2)	42 (0·7)	7 (0·1)
NAI (regimen unknown)^h^	1 (0·02)	5 (0·2)	17 (0·3)	82 (0·9)
NAI and non‐NAI^h^	75 (1·4)	15 (0·5)	76 (1·2)	18 (0·2)
NAI combination therapy^h^	134 (2·5)	23 (0·8)	144 (2·3)	71 (0·8)
Early NAI (≤2 days of symptom onset)^h^	1067 (19·8)	1057 (37·6)	1353 (21·4)	3459 (37·4)
Later NAI (>2 days after symptom onset)^h^	2843 (52·7)	998 (35·5)	3362 (53·1)	3221 (34·8)
Other in‐hospital treatment				
Antibiotics	3604 (60·3)	1731 (51·7)	4265 (60·5)	5521 (40·7)
Corticosteroids	1658 (27·7)	626 (18·7)	1709 (24·2)	1024 (7·5)
Hospital length of stay, days, median (IQR)	9 (5–17)	5 (3–7)	8 (4–17)	4 (2–7)
Other patient outcomes				
Acute respiratory distress syndrome	265 (4·4)	10 (0·3)	341 (4·8)	43 (0·3)
Ventilation support	2372 (39·7)	450 (13·4)	2619 (37·1)	1059 (7·8)
Admission to critical care	3335 (55·8)	764 (22·8)	3859 (54·7)	1989 (14·7)
Mortality	903 (15·1)	90 (2·7)	1014 (14·4)	496 (3·7)

a Any pneumonia includes influenza‐related pneumonia (IRP) (*n* = 5978) and physician‐diagnosed pneumonia (PDP) (*n* = 1076).

b No pneumonia includes no IRP (*n* = 3349), no PDP (*n* = 6616) and unknown pneumonia status (*n* = 3615).

c All percentages have been calculated using these denominators unless otherwise specified.

d Reported as clinically obese or using WHO definition for obesity (BMI ≥30 kg/m^2^ in adults aged ≥20 years).

e Proportions were calculated as a percentage of pregnant patients among female patients of reproductive age (13–54 years); the broader age range was selected in preference to the WHO definition (15–44 years) after consultation with data contributors to reflect the actual fertility experience of the sample.

f For definition of comorbidity, see Table S3.

g Denominators for pandemic vaccine based on patients admitted after 1 October 2009 (when vaccine potentially became available).

h Percentages calculated as a proportion of the total patients in that category who received neuraminidase inhibitor (NAI) therapy.

Overall, patients with IRP were more likely than patients with no IRP to be adult (*P* < 0·001), non‐pregnant (*P* < 0·001), free of underlying medical conditions (*P* = 0·038), be from outside the WHO European region (*P* < 0·001) and have laboratory‐confirmed influenza A(H1N1)pdm09 infection (*P* < 0·001). They were more likely to receive NAI treatment (*P* < 0·001), antibiotics (*P* < 0·001) and corticosteroids (*P* < 0·001), be admitted to critical care facilities (*P* < 0·001) and require ventilatory support (<0·001) or die (*P* < 0·001) (Table 1).

### Association between NAI treatment and IRP

Overall, 63 data sets provided data on 9327 hospitalised patients with a positive or negative diagnosis of pneumonia confirmed by chest radiography. After the exclusion of 14 data sets in which all patients had IRP (*n* = 1352, Table S5), 7975 patients remained in the analysis.

#### Early NAI (≤2 days) versus no NAI treatment

**Figure 2:**
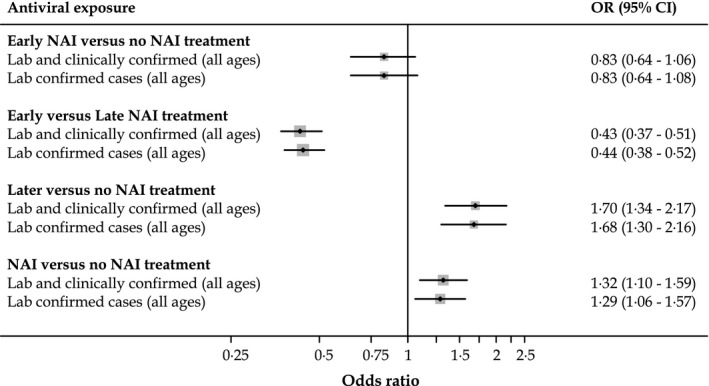
Summary of main findings for influenza‐related pneumonia (IRP) in laboratory‐ and clinical diagnosed influenza patients, all ages.

Early NAI use compared with no NAI use was not significantly associated with IRP in our overall sample [adjusted OR 0·83 (95% CI 0·64–1·06)], nor when we considered laboratory‐confirmed cases, adults, pregnant women or children (Table 2 and Figure 2). However, point estimates for subgroups tended to suggest an OR below unity, except in ICU patients. When considering ‘any pneumonia’, we found a borderline significant reduced OR associated with early NAI use in all patients [adjusted OR 0·83 (95% CI 0·70–0·98)], with further borderline significant risk reductions also noted among laboratory‐confirmed cases; these findings lost a statistical significance when further stratified by patient subgroups but the point estimates remained consistent (Table 2).

**Table 2:** Association between NAI treatment and pneumonia

Subgroups	Influenza‐related pneumonia (IRP)		Any pneumonia^a^	
	Crude OR (95% CI)	Adjusted^b^ OR (95% CI)	Crude OR (95% CI)	Adjusted^b^ OR (95% CI)
Early NAI (≤2 days) versus no NAI treatment				
Laboratory and clinically confirmed (all ages) (*n*_1_ = 2605; *n*_2_ = 6710)	0·97 (0·77–1·23)	0·83 (0·64–1·06)	1·02 (0·87–1·19)	0·83 (0·70–0·98)*
Laboratory‐confirmed cases (all ages) (*n*_1_ = 2462; *n*_2_ = 6541)	0·97 (0·76–1·24)	0·83 (0·64–1·08)	1·02 (0·87–1·19)	0·84 (0·70–0·99)*
Adults (≥16 years) (*n*_1_ = 1934; *n*_2_ = 3897)	0·90 (0·68–1·17)	0·80 (0·60–1·06)	1·00 (0·82–1·23)	0·82 (0·66–1·02)
Children (<16 years) (*n*_1_ = 670; *n*_2_ = 2765)	1·04 (0·61–1·77)	0·76 (0·42–1·36)	0·89 (0·69–1·14)	0·78 (0·59–1·03)
Pregnant (13–54 years) (*n*_1_ = 130; *n*_2_ = 424)	0·88 (0·27–2·93)	0·96 (0·29–3·20)	0·94 (0·41–2·18)	0·67 (0·26–1·76)
Intensive care unit (ICU) patients (all ages)				
Adults (≥16 years) (*n*_1_ = 583; *n*_2_ = 1015)	1·19 (0·67–2·13)	1·09 (0·59–2·02)	1·13 (0·76–1·67)	1·04 (0·69–1·56)
Children (<16 years) (*n*_1_ = 197; *n*_2_ = 447)	1·51 (0·58–3·97)	1·33 (0·46–3·78)	1·75 (0·99–3·12)	1·44 (0·79–2·62)
Early NAI (≤2 days) versus later NAI (>2 days)				
Laboratory and clinically confirmed (all ages) (*n*_1_ = 5058; *n*_2_ = 10 925)	0·34 (0·30–0·39)***	0·43 (0·37–0·51)***	0·40 (0·37–0·45)***	0·51 (0·46–0·57)***
Laboratory‐confirmed cases (all ages) (*n*_1_ = 4834; *n*_2_ = 10 667)	0·35 (0·30–0·40)***	0·44 (0·38–0·52)***	0·41 (0·37–0·45)***	0·52 (0·47–0·58)***
Adults (≥16 years) (*n*_1_ = 4189; *n*_2_ = 7549)	0·34 (0·29–0·39)***	0·43 (0·36–0·51)***	0·41 (0·36–0·46)***	0·51 (0·45–0·58)***
Children (<16 years) (*n*_1_ = 864; *n*_2_ = 3295)	0·43 (0·29–0·62)***	0·47 (0·32–0·71)***	0·43 (0·35–0·53)***	0·53 (0·43–0·66)***
Pregnant (13–54 years) (*n*_1_ = 256; *n*_2_ = 649)	0·26 (0·13–0·53)***	0·32 (0·13–0·75)**	0·27 (0·17–0·44)***	0·34 (0·20–0·58)***
ICU patients (all ages)				
Adults (≥16 years) (*n*_1_ = 1846; *n*_2_ = 2850)	0·38 (0·29–0·51)***	0·47 (0·34–0·63)***	0·55 (0·45–0·68)***	0·62 (0·50–0·77)***
Children (<16 years) (*n*_1_ = 251; *n*_2_ = 655)	0·46 (0·22–0·94)*	0·45 (0·20–1·01)	0·61 (0·42–0·89)**	0·71 (0·47–1·05)
Later (>2 days) versus no NAI treatment				
Laboratory and clinically confirmed (all ages) (*n*_1_ = 3991; *n*_2_ = 8251)	2·53 (2·02–3·16)***	1·70 (1·34–2·17)***	2·41 (2·09–2·79)***	1·57 (1·34–1·84)***
Laboratory‐confirmed cases (all ages) (*n*_1_ = 3822; *n*_2_ = 8048)	2·51 (1·98–3·16)***	1·68 (1·30–2·16)***	2·38 (2·06–2·76)***	1·55 (1·32–1·82)***
Adults (≥16 years) (*n*_1_ = 3263; *n*_2_ = 5572)	2·29 (1·78–2·95)***	1·64 (1·25–2·16)***	2·30 (1·91–2·77)***	1·58 (1·29–1·92)***
Children (<16 years) (*n*_1_ = 724; *n*_2_ = 2598)	2·26 (1·28–3·99)**	1·68 (0·89–3·16)	1·99 (1·55–2·57)***	1·42 (1·08–1·87)**
Pregnant (13–54 years) (*n*_1_ = 186; *n*_2_ = 383)	2·21 (0·76–6·45)	1·60 (0·40–6·49)	2·86 (1·30–6·25)**	1·58 (0·61–4·09)
ICU patients				
Adults (≥16 years) (*n*_1_ = 1511; *n*_2_ = 2249)	2·35 (1·31–4·23)**	1·55 (0·83–2·89)	1·68 (1·15–2·46)**	1·47 (1·00–2·17)*
Children (<16 years) (*n*_1_ = 236; *n*_2_ = 518)	5·84 (1·50–22·75)*	4·25 (1·07–16·88)*	3·50 (1·90–6·46)***	2·63 (1·39–4·96)**
NAI anytime versus no NAI treatment				
Laboratory and clinically confirmed (all ages) (*n*_1_ = 7975; *n*_2_ = 20 164)	1·57 (1·32–1·86)***	1·32 (1·10–1·59)**	1·62 (1·45–1·81)***	1·22 (1·08–1·38)**
Laboratory‐confirmed cases (all ages) (*n*_1_ = 7620; *n*_2_ = 19 553)	1·55 (1·29–1·86)***	1·29 (1·06–1·57)*	1·58 (1·41–1·78)***	1·19 (1·05–1·35)**
Adults (≥16 years) (*n*_1_ = 5964; *n*_2_ = 13 247)	1·53 (1·24–1·91)***	1·30 (1·03–1·63)*	1·63 (1·40–1·89)***	1·24 (1·06–1·46)**
Children (<16 years) (*n*_1_ = 2005; *n*_2_ = 6760)	1·38 (1·00–1·90)*	1·30 (0·92–1·82)	1·41 (1·18–1·69)***	1·18 (0·97–1·43)
Pregnant (13–54 years) (*n*_1_ = 348; *n*_2_ = 1430)	1·48 (0·58–3·74)	1·03 (0·32–3·29)	1·74 (0·93–3·23)	1·08 (0·52–2·22)
ICU patients (all ages)				
Adults (≥16 years) (*n*_1_ = 2721; *n*_2_ = 4071)	2·02 (1·30–3·14)**	1·57 (1·00–2·48)*	1·58 (1·14–2·18)**	1·38 (1·00–1·92)*
Children (<16 years) (*n*_1_ = 970; *n*_2_ = 1579)	1·45 (0·89–2·38)	1·39 (0·85–2·29)	1·76 (1·22–2·53)**	1·59 (1·10–2·30)*

*n*_1_ = total number of patients included in IRP analysis; *n*_2_ = total number of patients included in ‘any pneumonia’ analysis.

*P < 0·05, **P < 0·01, ***P < 0·001.

a Influenza‐related pneumonia and physician‐diagnosed pneumonia.

b Adjusted for treatment propensity quintiles, corticosteroid use and antibiotic use.

For this exposure, we also looked at the impact of corticosteroids on the association between NAI treatment and IRP. A test for interaction between NAI treatment and corticosteroids did not show any significant interaction (*P*‐value: 0·275). Stratified analysis (by corticosteroid use) did not show any significant association between NAI use and IRP (Table S9).

#### Early NAI (≤2 days) versus later NAI (>2 days) treatment

Early NAI treatment compared with later was associated with significantly lower odds of IRP [adjusted OR, 0·43 (95% CI, 0·37–0·51)] (Table 2 and Figure 2). The odds ratios did not change substantially when only cases of laboratory‐confirmed influenza were considered (Table 2). Similarly, statistically significant lower odds of IRP were observed in adults aged 16 years or older, children aged 0–15 years, pregnant women and among adult patients admitted to critical care. However, there was no statistically significant association with IRP among children admitted to critical care (Table 2). The pattern of these findings in terms of direction and significance was similar when considering ‘any pneumonia’ (Table 2).

#### Later NAI (>2 days) versus no NAI treatment

Neuraminidase inhibitor treatment beyond 2 days of symptom onset compared with no NAI was associated with statistically significant higher odds of IRP [adjusted OR, 1·70 (95% CI, 1·34–2·17)]. Similar statistically significant associations were observed among cases of laboratory‐confirmed influenza, adults and critically ill children, but not among all children, pregnant women and critically ill adults. Likewise, with ‘any pneumonia’, the direction and statistical significance of these findings did not change (Table 2 and Figure 2).

#### NAI anytime versus no NAI treatment

After adjustment, the likelihood of IRP in patients treated with NAI (administered at any point after illness onset) was 1·32 (95% CI 1·10–1·59), compared with no NAI treatment (Table 2 and Figure 2). This OR did not change substantially when only patients with laboratory‐confirmed A(H1N1)pdm09 were included [adjusted OR 1·29 (95% CI 1·06–1·57)]. Similarly, we observed significantly higher odds of IRP associated with NAI antiviral use in adults and borderline significantly increased odds of IRP in adults admitted to an ICU. However, there was no significant association between NAI treatment and IRP in children aged 0–15 years, pregnant women and critically ill children. The pattern of these findings was not changed by considering ‘any pneumonia’, except in children admitted to critical care where we observed statistically significant higher odds of IRP for patients treated with an NAI (at any time).

*Post hoc* analyses on non‐ICU patients (all ages) are shown in Table S6; children's subgroups aged <5 years and 5–15 are shown in Tables S7 (all severities) and S8 (critically ill).

### Impact of NAI treatment on clinical outcomes among patients with pneumonia

We performed a further analysis, restricted to patients with IRP (*n* = 5978) (Table 3), and a sensitivity analysis by including ‘any pneumonia’ patients (*n* = 7054). Data sets in which all patients had IRP (*n* = 1352 patients, 14 data sets) were re‐added at this juncture.

**Table 3:** Association between neuraminidase inhibitor (NAI) treatment and clinical outcomes among patients with pneumonia

Clinical outcomes/exposures studied	Influenza‐related pneumonia (IRP)		Any pneumonia^a^	
	Crude OR (95% CI)	Adjusted^b^ OR (95% CI)	Crude OR (95% CI)	Adjusted^b^ OR (95% CI)
Admission to an intensive care unit				
Early versus no NAI (*n*_1_ = 1480; *n*_2_ = 1855)	1·51 (1·01–2·25)*	1·44 (0·94–2·18)	2·02 (1·44–2·83)***	1·81 (1·27–2·58)**
Early versus later NAI (*n*_1_ = 3905; *n*_2_ = 4709)	1·15 (0·94–1·39)	0·89 (0·71–1·11)	1·09 (0·92–1·29)	0·95 (0·79–1·14)
Later versus no NAI (*n*_1_ = 3255; *n*_2_ = 3864)	2·59 (1·85–3·61)***	2·43 (1·71–3·45)***	2·91 (2·16–3·91)***	2·66 (1·95–3·62)***
NAI versus no NAI (*n*_1_ = 5962; *n*_2_ = 6976)	1·69 (1·30–2·19)***	1·59 (1·21–2·09)**	1·96 (1·55–2·50)***	1·78 (1·38–2·28)***
Ventilation support				
Early versus no NAI (*n*_1_ = 1131; *n*_2_ = 1287)	1·12 (0·70–1·79)	1·17 (0·71–1·92)	1·24 (0·82–1·87)	1·13 (0·73–1·75)
Early versus later NAI (*n*_1_ = 3084; *n*_2_ = 3459)	0·69 (0·56–0·86)**	0·68 (0·54–0·85)**	0·74 (0·60–0·90)**	0·75 (0·61–0·93)**
Later versus no NAI (*n*_1_ = 2489; *n*_2_ = 2760)	2·31 (1·50–3·55)***	2·48 (1·57–3·92)***	2·18 (1·48–3·21)***	2·21 (1·47–3·32)***
NAI versus no NAI (*n*_1_ = 4739; *n*_2_ = 5182)	1·70 (1·25–2·30)**	1·67 (1·22–2·29)**	1·69 (1·27–2·25)***	1·59 (1·19–2·13)**
Acute respiratory distress syndrome				
Early versus no NAI (*n*_1_ = 454; *n*_2_ = 546)	1·14 (0·32–4·07)	1·98 (0·46–8·54)	2·26 (0·76–6·67)	2·98 (0·77–11·60)
Early versus later NAI (*n*_1_ = 1234; *n*_2_ = 1434)	0·54 (0·33–0·90)*	0·65 (0·38–1·11)	0·55 (0·37–0·83)**	0·61 (0·40–0·94)*
Later versus no NAI (*n*_1_ = 1032; *n*_2_ = 1178)	2·34 (0·98–5·55)	2·23 (0·90–5·54)	3·42 (1·50–7·82)**	3·21 (1·36–7·58)**
NAI versus no NAI (*n*_1_ = 1549; *n*_2_ = 1836)	1·99 (0·84–4·70)	2·13 (0·87–5·21)	3·06 (1·35–6·94)**	3·14 (1·37–7·29)**
Mortality				
Early versus no NAI (*n*_1_ = 1490; *n*_2_ = 1866)	0·61 (0·38–0·96)*	0·72 (0·44–1·17)	0·59 (0·39–0·89)*	0·62 (0·40–0·96)*
Early versus later NAI (*n*_1_ = 3906; *n*_2_ = 4711)	0·84 (0·67–1·04)	0·70 (0·55–0·88)**	0·77 (0·63–0·95)*	0·69 (0·56–0·86)**
Later versus no NAI (*n*_1_ = 3266; *n*_2_ = 3875)	1·05 (0·73–1·52)	1·18 (0·81–1·74)	1·06 (0·76–1·49)	1·13 (0·80–1·61)
NAI versus no NAI (*n*_1_ = 5974; *n*_2_ = 7050)	0·88 (0·66–1·18)	0·90 (0·67–1·22)	0·89 (0·69–1·17)	0·89 (0·67–1·17)

*n*_1_ = total number of patients included in IRP analysis; *n*_2_ = total number of patients included in any pneumonia analysis.

*P < 0·05, **P < 0·01, ***P < 0·001.

a Influenza‐related pneumonia and physician‐diagnosed pneumonia.

b Adjusted for treatment propensity quintiles, corticosteroid use and antibiotic use.

In the IRP cohort, we did not observe any statistically significant associations with clinical outcomes when early NAI treatment was compared with no NAI treatment; but for ‘any pneumonia’, we observed that early NAI treatment versus no NAI was associated with an increased likelihood of admission to an ICU [adjusted OR, 1·81 (95% CI, 1·27–2·58); *P* = 0·001], but a reduced likelihood of mortality [adj. OR, 0·62 (95% CI, 0·40–0·96); *P* = 0·032].

In patients with IRP, early NAI treatment compared to later NAI was associated with significantly lower odds of ventilatory support [adjusted OR, 0·68 (95% CI, 0·54–0·85); *P* = 0·001] and mortality [adjusted OR, 0·70 (95% CI, 0·55–0·88); *P* = 0·003]. These effects were similar and remained statistically significant for ‘any pneumonia’.

Later NAI treatment versus no NAI was significantly associated with increased likelihood of ICU admission and ventilatory support. The pattern of these findings in terms of direction and significance was unchanged when considering ‘any pneumonia’. Likewise, patients with IRP who received NAI at any time versus no NAI treatment were more likely to be admitted to an ICU [adj. OR, 1·59 (95% CI, 1·21–2·09), *P* = 0·001] and receive ventilatory support [adj. OR, 1·67 (95% CI, 1·22–2·29), *P* = 0·001].

## Discussion

The strengths of this study include having data on a large number of patients of all ages hospitalised with influenza A(H1N1)pdm09 virus infection (mainly laboratory confirmed) from different geographical regions worldwide. Given the practical and ethical constraints likely to be involved in conducting placebo‐controlled trials during pandemic periods, the use of large‐scale pooled observational data offers the best chance of producing meaningful results on the effect of NAIs on severe outcomes such as pneumonia.

Our definition of IRP, which required radiographic evidence of pneumonia, represents a conservative estimate of all cases of pneumonia as radiography was not routinely performed for every patient in all participating centres. We therefore also performed separate analyses, which included patients with PDP. Some patients with PDP would not have had pneumonia (false positives), and thus, we expect that the true effect estimates of the association of NAI with pneumonia and clinical outcomes probably fall somewhere between the values obtained in the analyses for IRP and ‘any pneumonia’.

However, there are inevitable limitations, based on the use of retrospective observational data. Because we found an increase in IRP in several comparisons where we might have expected NAIs to have a protective effect, this suggests that our propensity scoring was not able to fully adjust for the tendency to use NAIs in more severe disease. We were unable to fully adjust for severity of illness within each propensity score because the different severity measures used across individual data sets were disparate. Furthermore, we included a broad spectrum of pneumonia severity and the available data did not permit stratification according to pneumonia severity (e.g. using CURB65 or the Pneumonia Severity Index).

### NAI treatment and occurrence of pneumonia

Our findings that early initiation of NAI treatment (≤48 hours after illness onset) compared with later was associated with a significant reduction in IRP and ‘any pneumonia’ corroborate those previously reported from observational data on hospitalised influenza patients.^9^, ^17^, ^19^ These trends were consistently observed across multiple subgroups: laboratory‐confirmed influenza, adults, children, pregnant women and adults requiring critical care (but not children). For early treatment versus none, highly consistent, protective point estimates were also generated for most comparisons in adults and children, but failed to reach a statistical significance for IRP [possibly due to type II errors (sample size) although they reached borderline significance for ‘any pneumonia’ (all cases)]. As such, the results are somewhat incongruent with our previous work, which showed a 50% reduction in mortality associated with early treatment versus none.^18^ It is possibly a combination of residual confounding and misclassification of pneumonia that has led to our current results, and it remains plausible that these weak signals still suggest a reduction in the occurrence of IRP.

Our other findings that NAI treatment at any time versus no NAI, and later NAI treatment compared with no NAI, universally increased the risks of IRP, contrast sharply with previous observational data on hospitalised influenza patients which found that NAI treatment (irrespective of timing) and later antiviral therapy (initiated >48 hours after illness onset) may improve a range of clinical outcomes.^19^, ^23^, ^24^, ^25^, ^26^, ^27^, ^28^ Essentially similar observations were made for ‘any pneumonia’.

Thus, in terms of the occurrence of pneumonia, our data suggest differential effects depending on the timing and use of NAIs; apparent harm associated with any or later NAI use versus no NAI; but potential benefit from early NAI use versus late NAI use or none. Based upon what is known about the mechanism of action of NAIs,^29^, ^30^ it is theoretically possible that treatment might be ineffective [tending to produce an odds ratio (OR) close to 1] but rather implausible that it would be genuinely harmful, producing an OR > 1 as we measured. Instead, we surmise that NAIs were often prescribed after the development of pneumonia or clinical deterioration; furthermore, patients with IRP were admitted to hospital a median of 4 days from symptom onset, compared to 2 days for those with no pneumonia. A process of reverse causation is more likely to be responsible for the elevated risk of IRP associated with any or late NAI treatment versus none. Indeed, from our data set, we were able to record the timing of initiation of NAI treatment in relation to illness onset, but we lacked the ability to record the timing of treatment in relation to the development of pneumonia, which precluded us conducting a survival analysis. With regard to the severity of illness at the time of initiating NAI therapy, one functional measure would have been to consider site of NAI treatment initiation (outpatient, emergency department, hospital ward, ICU); unfortunately, we were not able to do this because overall there were too many missing data.

### NAI treatment and clinical outcomes in pneumonia

Our other main finding relates to the effect of NAI treatment on clinical outcomes in patients with IRP. Our data reveal that patients with IRP, who were treated early with an NAI versus later, experienced a roughly one‐third lower likelihood of dying or requiring ventilatory support. A mortality reduction of similar magnitude was noted when comparing early NAI versus no NAI, which was statistically significant for the analysis of ‘any pneumonia’, but not for IRP. Although we advise caution in the interpretation of these subgroup analyses, essentially the same finding has been made about ventilatory support in a very large cohort of children hospitalised with seasonal and pandemic influenza.^31^

We also found that among patients with ‘any pneumonia’, those who received NAIs were more likely to be managed in an ICU or require ventilatory support compared to those not treated with NAIs, regardless of the timing of treatment. Confounding by indication is an important consideration in relation to these data; that is, patients with severe pneumonia or ARDS who were escalated to ICU‐based care would be more likely to be preferentially treated with NAIs compared to those not requiring ICU; indeed, in the PRIDE data set overall (*n* = 29 259), we noted that 82% of ICU patients received an NAI compared with 61% in non‐ICU patients (*P* < 0·001). The alternative explanation that NAI treatment results in clinical deterioration with resultant increased requirements for ICU admission or ventilatory support, but no increase in mortality is unlikely and our results should not be used to justify the avoidance of early empirical use of NAIs for patients who are severely unwell with suspected influenza.

### Technical limitations

Insufficient data on influenza vaccination limited our ability to assess its potential effect on the clinical course of influenza A(H1N1)pdm09 virus infection, albeit that 9890 of 20 634 patients (48·5%) were admitted prior to November 2009 and could not have benefitted from H1N1pdm09 vaccine as it would not have been available by this point.

There were wide variations across included study centres in terms of individual study period, healthcare systems, clinical practice, treatment policies and resource availability. Although we attempted to control for these study‐level biases using generalised linear mixed models, residual confounding is possible. Likewise, we cannot completely eliminate misclassification of exposure, covariate or outcome variables. Notwithstanding, we attempted to account for misclassification bias by conservatively restricting our main analysis to IRP based on chest radiograph reports. But we were unable to discriminate between viral pneumonia, bacterial pneumonia and concurrent viral and bacterial pneumonia, nor differentiate between community‐ and hospital‐acquired pneumonia.

Despite requesting a minimum set of data variables (Table S2), the nature of the surveillance data sets provided, which were set up for monitoring during a public health emergency, meant that there were missing data on some variables of interest (e.g. admission diagnosis, comorbidities, interval from the onset of symptoms to NAI treatment, severity of disease at presentation, influenza vaccination, concomitant therapies, complications, information on follow‐up).

Finally, this study does not reflect the full spectrum of disease caused by influenza A(H1N1)pdm09 virus infection in the community as it only examined hospitalised patients.

### Implications and conclusions

Early NAI treatment probably reduces the likelihood of IRP. We observed highly consistent protective point estimates for early initiation of NAI treatment versus late and early treatment versus no NAI, but only the former was statistically significant; therefore, the evidence is strongest for an effect of early versus later NAI treatment. Overall, NAI treatment compared with no NAI treatment was associated with an increased likelihood of IRP; we surmise this because NAIs are sometimes started later in response to the development of pneumonia.

In patients with IRP, early NAI treatment versus later reduced the need for ventilatory support and subsequent mortality. Because randomised controlled trials of NAI treatment versus no NAI or placebo, or early NAI treatment versus late, are unlikely to be ethically or practically feasible, further evidence is needed from well‐designed, prospective cohort studies in which disease severity and the dates of symptom onset, hospital admission, NAI treatment initiation and pneumonia onset are all accurately and consistently described.

## Funding

The PRIDE study is funded via an unrestricted educational grant from F. Hoffmann‐La Roche, Switzerland [the manufacturers of Oseltamivir (Tamiflu^®^)]. The funder has had no role in protocol design, no opportunity to comment on it and no opportunity to see it other than via the PROSPERO website; no access to any data (and no rights to future access); no role in analysis or interpretation; no opportunity to preview results/findings before entry into the public domain; no opportunity to contribute to, preview or comment on manuscripts and presentations arising from this work. The research contract between the University of Nottingham and the funder is freely available for inspection (commercial details redacted) at: http://www.nottingham.ac.uk/research/groups/healthprotection/projects/pride.aspx.

## Conflict of interest

Puja R Myles and Jo Leonardi–Bee are recipients of grants from F. Hoffmann‐La Roche, during the conduct of the study. Wei Shen Lim reports unrestricted investigator‐initiated research funding from Pfizer to his department for research into pneumonia, a research grant from the National Institute for Health Research and consultancy fees paid to his department from the Wellcome Trust, outside the submitted work. Roland Bingisser has received consultancy fees from Philips and Alere. Jordi Carratala reports grants from Instituto de Salud Carlos III, personal fees from F. Hoffmann‐La Roche, outside the submitted work. Allison McGeer reports grants from Hoffman LaRoche, during the conduct of the study, grants from GlaxoSmithKline, grants from Crucell (Johnson & Johnson), other from Cepheid, grants from Merck, grants from Sanofi‐Pasteur, outside the submitted work. Barbara Rath reports grants from Hofmann LA Roche Inc. to her institution (Charité Universitätsmedizin Berlin), outside the submitted work. Dat Tran reports grants from Canadian Institutes of Health Research/SickKids Foundation New Investigator Grant XG08‐049R, grants from Canadian Institutes of Health Research Catalyst Grant CAT86860, grants from University of Toronto Dean's Fund Pilot Study Grant, during the conduct of the study. Wendy Vaudry reports grants from Canadian Pediatric Society, grants from Public Health Agency of Canada, during the conduct of the study. Jonathan S Nguyen‐Van‐Tam reports that a grant from F. Hoffmann‐La Roche funded the current study; grants from GlaxoSmith Kline in the area of influenza and non‐financial support from ESWI (European Scientific Working Group on Influenza) to lecture on influenza, outside the submitted work; his brother is a current employee of GlaxoSmithKline. All other named authors declare no conflict of interests. JSN‐V‐T is Editor‐in‐Chief of Influenza and Other Respiratory Viruses; however he played no role whatsoever in the editorial process for this paper, including decisions to send the manuscript for independent peer‐review or about final acceptance of a revised version. All of the above functions were handled independently by Dr Alan Hampson, Senior Editor (formerly Editor‐in‐Chief).

## Author contributions

JSN‐V‐T, PRM, WSL, JL‐B, SGM and SV conceived and designed the study. All authors, apart from SGM, SV, JL‐B and WSL, contributed to the acquisition and local preparation of constituent data sets. SGM, SV, PRM and JL‐B contributed to data set amalgamation and standardisation, design of statistical analyses and data analysis. JSN‐V‐T, PRM, JL‐B, WSL, SGM and SV interpreted the data and wrote the paper. All authors contributed to critical examination of the paper for important intellectual content and approval of the final report. Each author acts as the guarantor of data from their individual study centre; JSN‐V‐T and PRM act as overall guarantors for the pooled analysis and the report.

## Supporting information


**Figure S1.** Date of admission of 29 512 patients hospitalized with A(H1N1)pdm09 infection (by month).
**Table S1.** PRIDE study Investigators.
**Table S2.** Minimum dataset requirement.
**Table S3.** Standardised dataset – data dictionary with definitions used in this analysis.
**Table S4.** Comparison of hospitalised patients included in analysis compared with excluded patients.
**Table S5.** Characteristics of individual studies contributing to the current analysis.
**Table S6.** Sensitivity analysis excluding all ICU patients.
**Table S7.** Association between NAI treatment and pneumonia (all children).
**Table S8.** Association between NAI treatment and pneumonia (critically ill children).
**Table S9.** Stratified analysis based on steroid use.

## Appendix 1 PRIDE Consortium Investigators

Maria de Lourdes Aguiar‐Oliveira, Tarig SA Al Khuwaitir, Malakita Al Masri, Robed Amin, Elena Ballester‐Orcal, Jing Bao, Ariful Basher, Edgar Bautista, Barbara Bertisch, Julie Bettinger, Robert Booy, Emilio Bouza, Ilkay Bozkurt, Heinz Burgmann, Elvira Čeljuska‐Tošev, Kenny KC Chan, Yusheng Chen, Tserendorj Chinbayar, Catia Cilloniz, Rebecca J Cox, Elena B Sarrouf, Wei Cui, Simin Dashti‐Khavidaki, Bin Du, Hicham El Rhaffouli, Hernan Escobar, Agnieszka Florek‐Michalska, John Gerrard, Stuart Gormley, Sandra Götberg, Behnam Honarvar, Jianming Hu, Christoph Kemen, Evelyn SC Koay, Miroslav Kojic, Koichiro Kudo, Win M Kyaw, Leonard Leibovici, Xiao‐li Li, Hongru Li, Romina Libster, Tze P Loh, Deborough Macbeth, Efstratios Maltezos, Débora N Marcone, Magdalena Marczynska, Fabiane P Mastalir, Auks≐ Mickiene, Mohsen Moghadami, Lilian Moriconi, Maria E Oliva, Blaž Pečavar, Philippe G Poliquin, Mahmudur Rahman, Alberto Rascon‐Pacheco, Samir Refaey, Brunhilde Schweiger, Anna C Seale, Bunyamin Sertogullarindan, Fang G Smith, Ayper Somer, Thiago ML Souza, Frank Stephan, Payam Tabarsi, CB Tripathi, Diego Viasus, Qin Yu, Wei Zhang, Wei Zuo.
