# Supplementary material for: Impact of neuraminidase inhibitors on influenza A(H1N1)pdm09‐related pneumonia: an individual participant data meta‐analysis
Source: Influenza Other Respir Viruses. 2016 Feb 1;10(3):192–204. doi: 10.1111/irv.12363 (PMC4814862; doi:10.1111/irv.12363)
Supplement: Supplementary file 1 — Figure S1. Date of admission of 29 512 patients hospitalized with A(H1N1)pdm09 infection (by month). Table S1. PRIDE study Investigators. Table S2. Minimum dataset requirement. Table S3. Standardised dataset – data dictionary with definitions used in this analysis. Table S4. Comparison of hospitalised patients included in analysis compared with excluded patients. Table S5. Characteristics of individual studies contributing to the current analysis. Table S6. Sensitivity analysis excluding all ICU patients. Table S7. Association between NAI treatment and pneumonia (all children). Table S8. Association between NAI treatment and pneumonia (critically ill children). Table S9. Stratified analysis based on steroid use. [file IRV-10-192-s001.docx]

**Impact of neuraminidase inhibitors on influenza A(H1N1)pdm09-related pneumonia: an IPD meta-analysis**

**Online Data Supplement**

Table E1: PRIDE study Investigators

| **Study no†** | **Authors** | **Author Affiliation** |
| --- | --- | --- |
| 1§ | YS Leo, WM Kyaw | Department of Infectious Diseases, Tan Tock Seng Hospital, Singapore |
| 2 | A Al Mamun ^1^, M Rahman ^2^, E Azziz-Baumgartner ^3^ | ^1^ International Centre for Diarrhoeal Diseases  Research Bangladesh (ICDDRB), Dhaka, Bangladesh  ^2^ Institute of Epidemiology, Disease Control and Research (IEDCR), Dhaka, Bangladesh  ^3^ Centers for Disease Control and Prevention, Atlanta, Georgia, USA |
| 3 | A Moradi ^1 2^, P Tabarsi ^2^ | ^1^ The Division of Ocular Immunology, Department of Ophthalmology, Johns Hopkins University School of Medicine, Baltimore, Maryland, United States  ^2^ National Research Institute for Tuberculosis and Lung Disease, Massih Daneshvari Hospital, Shahid Beheshti University of Medical Sciences, Tehran, Iran |
| 4 | A H Rodríguez* for the H1N1 Sociedad Española de Medicina Intensiva, Crítica y Unidades Coronarias (SEMICYUC) working group | *Hospital Joan XXIII, Critical Care Department - IISPV - URV - CIBERES, Tarragona, Spain |
| 5 | AL Higuera Iglesias ^1^ , E Bautista ^2^ | ^1^ Epidemiology Research Unit, Instituto Nacional de Enfermedades Respiratorias, Ismael Cosío Villegas, Mexico City, Mexico  ^2^ Critical Care Department, Instituto Nacional de Enfermedades Respiratorias, Ismael Cosío Villegas, Mexico City, Mexico |
| 6§ | J Fraser ^1^, AC Seale ^1 2^ | ^1^ Paediatric Intensive Care Unit, Bristol Children's Hospital, UK  ^2^ Bristol Children’s Vaccine Centre, School of Clinical Sciences, University of Bristol, Bristol, UK |
| 7 | A van Zwol | Department of Pediatric Intensive Care, VU University Medical Center, Amsterdam, Netherlands |
| 8 | AMC Kwan, KKC Chan | Department of Intensive Care, Pamela Youde Nethersole Eastern Hospital, Chai Wan, Hong Kong |
| 9 | D Velyvyte, A Mickiene | Lithuanian University of Health Sciences, Kaunas, Lithuania |
| 10 | M Hoffmann^1^ B Bertisch^2^ | ^1^Division of Infectious Diseases and Hospital Epidemiology, Kantonsspital St. Gallen, Switzerland  ^2^University of Bern, Institute of Social and Preventive Medicine (ISPM), Finkenhubelweg, Bern, Switzerland |
| 11 | B A Rath^1^, B Schweiger^2^ | ^1^ Department of Pediatrics, Division of Pneumonology-Immunology, Charité University Medical Center, Berlin, Germany  ^2^ National Reference Centre Influenza at Robert Koch Institute, Berlin, Germany |
| 12 | B Cao, X Li | Beijing Chao-Yang Hospital, Capital Medical University, Beijing, China |
| 13 | X Hu, B Du | Peking Union Medical College Hospital, Beijing, China |
| 14 | B Ozbay, B Sertogullarindan | Yuzuncu Yil University Medical Faculty, Department of Pulmonary Medicine Van, Turkey |
| 15 | C Bantar, ME Oliva | Dept. of Infection Control, Hospital San Martín de Paraná, Entre Ríos, Argentina |
| 16 | A Torres, C Cilloniz | Hospital Clinic, University of Barcelona IDIBAPS, CIBERES, Spain |
| 17 | H Khalili, S Dashti-Khavidaki, | Department of Clinical Pharmacy, Faculty of Pharmacy, Tehran University of Medical Sciences, Tehran, Iran |
| 18 | D Tran | Division of Infectious Diseases, Department of Paediatrics, The Hospital for Sick Children  University of Toronto, Canada |
| 19 | E Langenegger | Department of Obstetrics and Gynaecology, Stellenbosch University and Tygerberg, South Africa |
| 20 | EB Sarrouf ^1^, MR Cuezzo ^1^, E Azziz-Baumgartner ^2^ | ^1^ Ministerio de Salud de Tucumán, Argentina  ^2^ Centers for Disease Control and Prevention, Atlanta, Georgia, USA |
| 21 | E Talarek, M Marczynska | Department of Children`s Infectious Diseases, Medical University of Warsaw, Poland |
| 22 | F Madanat | King Hussein Cancer Center, Department of Pediatrics, Amman , Jordan |
| 23 | G Dubnov-Raz | The Edmond and Lily Safra Children's Hospital, Sheba Medical Center, Israel |
| 24 | G Keijzers, J Gerrard, D Macbeth | Gold Coast Hospital, Gold Coast, Australia |
| 25 | G Metan, I Bozkurt | Department of Infectious diseases and clinical microbiology, Erciyes University Faculty of Medicine, Kayseri, Turkey |
| 26 | I Lahlou Amine, H El Rhaffouli | University Mohammed V-Souissi, Faculty of Medicine and Pharmacy, Mohammed V Military Teaching Hospital, Biosafety Level 3 and Research Laboratory, Rabat, Morocco |
| 27 | I Bonmarin | Institut de Veille Sanitaire, France |
| 28 | J Carratala*, D Viasus* for the Novel Influenza A (H1N1) Study Group of the Spanish Network for Research in Infectious Diseases (REIPI) | *Department of Infectious Diseases, Hospital Universitari de Bellvitge-IDIBELL, L'Hospitalet de Llobregat, REIPI, University of Barcelona, Barcelona, Spain |
| 29 | JW Tang ^1 2 3^, TP Loh ^1^, ESC Koay ^1 4^ | ^1^ Molecular Diagnostic Centre, Department of Laboratory Medicine National University Hospital, Singapore  ^2^ Alberta Provincial Laboratory for Public Health, University of Alberta Hospital, Canada  ^3^ Department of Medical Microbiology and Immunology, University of Alberta, Edmonton, Alberta, Canada  ^4^ Department of Pathology, National University of Singapore, Singapore |
| 30 | W Vaudry* (for the Canadian Immunization Monitoring Program, Active [IMPACT]), J Bettinger ^1^, D Tran ^2^ | ^1^ Vaccine Evaluation Center, BC Children’s Hospital and the University of British Columbia, Pediatrics, Vancouver, BC, Canada  * Division of Infectious Diseases, Department of Pediatrics, University of Alberta, Stollery Children’s Hospital, Edmonton, Alberta, Canada  ^2^ Division of Infectious Diseases, Department of Paediatrics, The Hospital for Sick Children  University of Toronto, Canada |
| 31 | JT Denholm | Victorian Infectious Diseases Service and Department of Microbiology and Immunology, at the Peter Doherty Institute for Infection and Immunity, Parkville, Australia. |
| 32 | G Kusznierz ^1^, H Escobar ^2^, E Azziz-Baumgartner ^3^ | ^1^ National Institute of Respiratory Diseases "Emilio Coni" ANLIS "C. Malbran, Argentina  ^2^ Ministry of Health of the province of Santa Fe, Argentina  ^3^ Centers for Disease Control and Prevention, Atlanta, Georgia, USA |
| 33 | C Báez ^1^, L Moriconi ^1^, E Azziz-Baumgartner ^2^ | ^1^ Ministerio de Salud de la Provincia de Buenos Aires, Argentina  ^2^ Centers for Disease Control and Prevention, Atlanta, Georgia, USA |
| 34 | M Giannella, E Bouza | Microbiología Clínica y Enfermedades Infecciosas, Hospital General Universitario Gregorio Marañón, Departamento de Medicina, Universidad Complutense de Madrid, Madrid, Spain |
| 35 | M Echavarria, DN Marcone | Clinical Virology Laboratory, CEMIC University Hospital, Galvan 4102, (1431) Buenos Aires, Argentina |
| 36§ | M Knight* for the UK Obstetric Surveillance System (UKOSS) | * National Perinatal Epidemiology Unit, Nuffield Department of Population Health, University of Oxford, UK |
| 37 | M Bassetti | Santa Maria Misericordia Hospital, Udine Italy |
| 38 | M Ozkan | Dr. Sami Ulus Research and Training Hospital of Women's and Children's Health and Diseases, Clinic of Pediatric Neurology, Ankara, Turkey |
| 39 | M Paul^1^, L Leibovici^2^ | ^1^Division of Infectious Diseases, Rambam Health Care Campus, Haifa, Israel  ^2^Medicine E, Rabin Medical Center, Beilinson Hospital, Petah Tikva, Israel |
| 40 | MF Jiménez, FP Mastalir | Departamento de Ginecologia e Obstetrícia - UFCSPA, Preceptora da Residência Médica do Hospital Fêmina, Brazil |
| 41 | P Gérardin | ^1^ NICU/PICU, PFME, CHU Saint Pierre -  ^2^ CIC 1410 (CHU/Inserm/ University of La Réunion/URML-OI), CHU Saint Pierre -  ^3^ UMR PIMIT (CHU/Inserm/University of La Réunion/IRD/ CNRS), CYROI, Saint Denis - Reunion island, France |
| 42§ | P Zarogoulidis, E Maltezos, | Unit of Infectious Diseases, University General Hospital of Alexandroupolis, Democritus University Thrace, Dragana, Greece |
| 43§ | PAD Duarte | Universidade Estadual do Oeste do  Paraná - UNIOESTE - Cascavel (PR), Brazil |
| 44 | WN Araújo | University of Brasília, Brazil |
| 45 | R Linko* for the FINNH1N1-study group | *Helsinki University Hospital, Helsinki, Finland |
| 46§ | A Kandeel, S Refaey | Ministry of Health in Egypt, Cairo, Egypt |
| 47 | SR Dominguez | Department of Pediatric Infectious Diseases, Children's Hospital Colorado, University of Colorado School of Medicine, Aurora, Colorado, USA |
| 48 | SH Törün, A Somer | Department of Pediatric Infectious Diseases, Istanbul University Istanbul Medical Faculty, Istanbul, Turkey |
| 49 | ST Fanella, G Poliquin | Section of Pediatric Infectious Diseases, University of Manitoba, Winnipeg, Manitoba, Canada |
| 50 | S Gubbels | Department of Infectious Disease Epidemiology, Sector for National Health Documentation and Research, Statens Serum Institut, Copenhagen, Denmark |
| 51 | MM Barhoush, TSA Al Khuwaitir, | Department of Medicine, King Saud Medical City, Riyadh, Kingdom of Saudi Arabia |
| 52 | MM Siqueira^1^, TML Souza^2^, ML Aguiar-Oliveira^1^ | ^1^ Laboratory of Respiratory Viruses, Oswaldo Cruz Institute/Fiocruz, Rio de Janeiro, Brazil  ^2^ Centro de Desenvolvimento Tecnológico em Saúde (CDTS) and Laboratório de imunofarmacologia, Instituto Oswaldo Cruz - Fiocruz, Rio de Janeiro, Brazil |
| 53 | T Vidmar | General Hospital Slovenj Gradec, Slovenia |
| 54 | T Manabe ^1^ , K Kudo ^2^ | ^1^ Graduate School of Comprehensive Human Sciences, University of Tsukuba, Tsukuba, Ibaraki, Japan  ^2^ National Center for Global Health and Medicine, Tokyo Japan |
| 55 | AP Anovadiya, CB Tripathi | Department of Pharmacology, Government Medical College and Sir Takhtsinhji General Hospital, Bhavnagar, Gujarat, India |
| 56§ | VH Borja-Aburto, A Rascon-Pacheco | Instituto Mexicano del Seguro Social (IMSS), Mexico |
| 57 | P Liu, W Cui | Department of Infectious Diseases, the First Affiliated Hospital, China Medical University, Shenyang, China |
| 58 | I Kuzman, E Čeljuska-Tošev | University Hospital for Infectious Diseases, University of Zagreb, School of Medicine, Zagreb, Croatia |
| 59 | JS Nguyen Van-Tam* for the Influenza Clinical Information Network (FLU-CIN) | *Division of Epidemiology and Public Health, University of Nottingham, Nottingham, UK |
| 60 | J Skręt-Magierło ^1^, A Florek-Michalska ^2^ | ^1^ Uniwersytet Rzeszowski, Poland  ^2^ Kliniczny Oddzial Ginekologii i Poloznictwa, WSS Rzeszow, Poland |
| 61 | B Beovic, B Pecavar | Department of Infectious Diseases, University Medical Centre, Ljubljana, Slovenia |
| 62 | D Mikic, M Kojic | Military Medical Academy, Clinic for Infectious and Tropical Diseases, Serbia. |
| 63 | D Parekh, FG Smith | Perioperative, Critical Care and Trauma Trials Group, School of Clinical and Experimental Medicine, University of Birmingham, UK |
| 64 | FP Polack^1 2^, R Libster^1 2 3^ | ^1^ Vanderbilt Vaccine Center, Department of Pediatrics, Vanderbilt University, Nashville, TN, USA  ^2^ Fundacion INFANT, Buenos Aires, Argentina  ^3^ National Scientific and Technical Research Council – Argentina (CONICET) |
| 65 | Z Gao ^1^, J Bao ^1^, Y Chen ^2^, H Li ^2^, Q Yu ^3^, J Hu ^3^, W Zhang ^4^, W Zuo ^4^ | ^1^ Department of Respiratory & Critical Care Medicine, Peking University People's Hospital, Beijing, China  ^2^ Respiratory Department of Fujian Provincial  Hospital ，Fujian, China  ^3^ Respiratory Department of The First Affiliated Hospital of Lanzhou University, Lanzhou, China  ^4^ Respiratory Department of The First Affiliated Hospital of Nanchang University, Jiangxi, China |
| 66 | H Burgmann, W Poeppl | Medical University of Vienna, Austria |
| 67 | KB Lankarani, B Honarvar | Health Policy Research Center, Shiraz University of Medical Sciences, Shiraz, Iran |
|  | M Moghadami | HIV/AIDS Research Center ,Shiraz University of Medical Sciences, Shiraz, Iran |
| 68 | P Nymadawa, T Chinbayar | National Influenza Center, National Center of Communicable Diseases, Ministry of Health, Ulaanbaatar, Mongolia |
| 69 | PH Hoger, C Kemen, S Götberg | Cath. Children´s Hospital Wilhelmstift, Liliencronstr, Hamburg, Germany |
| 70 | G Khandaker, R Booy | National Centre for Immunisation Research and Surveillance (NCIRS), The Children’s Hospital at Westmead, University of Sydney, New South Wales, Australia |
| 71 | Z Memish, M al Masri | Ministry of Health, Riyadh, Kingdom of Saudi Arabia |
| 72§ | QT Islam, A Basher, R Amin | Dhaka Medical College Hospital, Bangladesh |
| 73 | TM Uyeki* for the National Heart, Lung  Blood Institute, ARDSNET Clinical Trials (NHLBI ARDS) Network | *Influenza Division, National Center for Immunization and Respiratory Diseases, Centers for Disease Control and Prevention, Atlanta, USA |
| 74 | TM Uyeki* for the Pediatric Acute Lung  Injury and Sepsis Investigator's (PALISI) Network | *Influenza Division, National Center for Immunization and Respiratory Diseases, Centers for Disease Control and Prevention, Atlanta, USA |
| 75 | J Herberg, S Gormley | Section of Paediatrics, Division of Infectious Disease, Imperial College, London, UK |
| 76 | A McGeer | Toronto Invasive Bacterial Diseases Network, Toronto, Canada |
| 77 | KGI Mohn ^1 2^, RJ Cox ^1 2 3^ | ^1^ Section for Infectious Diseases, Medical Department, and Department of Research and Development, Haukeland University Hospital ^2^ The Influenza Centre, Department of Clinical Science, University of Bergen, Norway  ^3^ K.G. Jebsen Centre for research on Influenza Vaccines, University of Bergen, Norway |
| 78‡ | E Mayo-Montero, E Ballester-Orcal | Instituto de Medicina Preventiva de la Defensa, Capitan Medico Ramon y Cajal (IMPDEF). Ministerio de Defensa, Madrid, Spain |
| 79‡ | R Bingisser, F Stephan | Department of Emergency Medicine, University Hospital Basel, Switzerland |

*Named author

† Baseline characteristics of each constituent dataset included in the current analysis are presented in Supplementary Appendix 4.

§ Centres not included in the current analysis as they lacked data on pneumonia status.

‡ Centres not included in the current analysis as they include only outpatient data.

**Table E2: Minimum dataset requirement**

| **Data variables** | **Description/definition** |
| --- | --- |
| **About the data** | |
| 1. Source of data | E.g. hospital or emergency department, community/ GP sentinel data, national surveillance data etc. |
| 1. Patient identifier | A unique identifier for each patient e.g. a specific number that corresponds to each patient |
| **Patient characteristics** | |
| 1. Age | Date of birth/ age at hospital admission |
| 1. Sex | Male/ female |
| 1. Any other risk factors | Such as obesity/ BMI, smoking status etc. |
| 1. Seasonal influenza vaccination status | Yes/no |
| **Clinical characteristics** | |
| 1. Underlying Co-morbidities 2. Asthma 3. COPD 4. Pneumonia 5. Other pulmonary diseases 6. Other chronic conditions | List of underlying medical conditions for each individual patient |
| 1. Pregnancy | Pregnancy/ fetal wellbeing assessments e.g. weeks of gestation including pregnancy outcome |
| 1. Clinical examinations 2. Influenza presenting symptoms on hospital admission 3. Admission laboratory examinations performed 4. Chest x-ray examination | - Date of symptoms onset - List of symptoms presenting at hospital admission - E.g. technique used for laboratory confirmation of influenza AH1N1 e.g. RT-PCR, viral culture, etc. - Date chest x-ray taken - Findings upon x-ray examination |
| **Treatment characteristics** | |
| 1. In-hospital antiviral treatment (oseltamivir, zanamivir etc.) | - Date of prescription/treatment including name of antiviral agent (i.e. oseltamivir/ zanamivir), route of administration(e.g. inhaled, oral , IV), dosage and dose frequency, treatment duration |
| 1. In-hospital antibiotics treatment | - List of names of administered antibiotics including route(e.g. inhaled, oral , IV), dose and dose frequency, duration and dates prescribed |
| 1. Other in-hospital additional treatment therapies e.g. corticosteroids, antipyretics etc. | - List of names of other administered treatments including route(e.g. inhaled, oral , IV), dose and dose frequency, duration and dates prescribed |
| **Outcome** (please provide data on at least one of the outcome measures) | |
| 1. Hospitalisation | - Dates of admission and discharge - Primary admitting diagnosis for cases admitted to hospital - Secondary diagnoses |
| 1. Pneumonia | - Yes/ no, Unilateral or bilateral etc. |
| 1. Admission to critical care facilities i.e. ICU and/ or HDU | - Specific dates of transfer and discharge to/ from the HDU or ICU - Primary cause of transfer to HDU/ ICU - Need for invasive mechanical ventilation (MV) including non-invasive mechanical ventilation(NIV) and ECMO - Other severity scores e.g. duration MV, NIV, ECMO |
| 1. Mortality in hospital | - Date of death - Primary cause of death |

**Table E3: Standardised dataset - data dictionary with definitions used in this analysis**

| **Variable name in Stata** | **Description** | **Coding** |
| --- | --- | --- |
| **About the data** | | |
| study_group_id | Study group identifier (lookup codes in MS Excel metadata file) | Auto-numbering (_n); string |
| Patid | Study specific patient ID as provided by study groups; this will only be retained in the individual datasets but dropped from the pooled dataset | string |
| auto_patid | Auto-numbered patient ID generated for each study group dataset; this will only be retained in the individual datasets but dropped from the pooled dataset | Auto-numbering (_n) |
| pride_patid | Unique patient identifier created by concatenation of study_group_id and auto-numbered patient id (auto_patid) | String |
| data_source | Whether hospital, community or ICU | 1=hospital  2= ICU  3= community |
| Country | Country identifier | String |
| **Patient characteristics** | | |
| age_years | Age (at admission) in years; continuous variable | Place all under 1s in one category (replace <1s as ‘0’) |
| sex | gender | 1=male  0=female |
| obese | Either clinically recorded obesity or derived from BMI (BMI>30) | 1= yes  0= no |
| smoking | Current smoking status (ex and non-smokers will be considered one category) | 1= yes  0= no |
| pregnant | Pregnancy  (for sub-group analyses including pregnant women, only women of child-bearing age (13-54 years) will be considered) | 1= yes  0= no  (no separate code for ‘not applicable’; men and women not of child-bearing age will be dropped from any analyses including pregnant women) |
| comorbidity | Whether any co-existing comorbidity was present (either as recorded or derived on the basis of a record of one of the following comorbidities: asthma, COPD, other chronic lung disease, heart disease, cerebrovascular disease (not including uncomplicated hypertension), chronic liver disease, chronic renal disease, diabetes, neurological disease (including neurodevelopmental disorders), lymphoma, leukaemia, other malignancy, immunosuppression)  Note: pregnancy will not be considered a ‘comorbidity’ and nor will obesity | 1= any comorbidity  0= no recorded comorbidity |
| asthma | Asthma as recorded | 1= yes  0= no |
| copd | COPD as recorded | 1= yes  0= no |
| other_lung_ds | Chronic pulmonary diseases (other than asthma or COPD) | 1= yes  0= no |
| heart_ds | Chronic heart disease as recorded (includes congenital heart disease, hypertension with cardiac complications, chronic heart failure, individuals requiring regular medication and/or follow-up for ischaemic heart disease) | 1= yes  0= no |
| renal_ds | Chronic kidney disease (CKD) as recorded including CKD at stage 3, 4 or 5, chronic kidney failure, nephrotic syndrome, kidney transplantation. | 1= yes  0= no |
| liver_ds | Chronic liver disease as recorded (including cirrhosis, biliary atresia, chronic hepatitis) | 1= yes  0= no |
| cerebrovascular_ds | Cerebrovascular disease as recorded (including stroke, transient ischaemic attack but not including uncomplicated hypertension) | 1= yes  0= no |
| neurological_ds | Neurological disease as recorded (including neurodevelopmental disorders) | 1= yes  0= no |
| diabetes | Diabetes as recorded (including Type 1 diabetes, type 2 diabetes requiring insulin or oral hypoglycaemic drugs, diet controlled diabetes.) | 1= yes  0= no |
| immunosuppression | Immunosuppression due to disease or treatment. Includes patients undergoing chemotherapy leading to immunosuppression; asplenia or splenic dysfunction and HIV infection at all stages. | 1=yes  0= no |
| pdm09_flu_vac | H1N1pdm09 vaccination | 1= yes  0= no |
| **clinical characteristics** | | |
| onset_date | Date of onset of influenza like illness (ILI) | Recorded as date dd/mm/yy |
| admission_date | Date of first admission to hospital (whether to a general ward, HDU or ICU) | Recorded as date dd/mm/yy |
| time_to_admission | Time (in days) from onset of symptoms to hospital admission | Recorded as number of days |
| symptom_score | Severity of illness on presentation to hospital; non-weighted score derived by assigning a score of 1 for each of the following symptoms and then adding these together (myalgia; malaise; headache; sore throat; cough; nasal symptoms including runny nose, stuffy nose, sneezing; diarrhoea/vomiting)  Note: fever and shortness of breath will be considered as separate variables to emphasise greater weighting of these symptoms | Recorded as score (maximum score of 7 possible) |
| fever | Fever on presentation (as recorded) | 1= yes  0= no |
| shortness_of_breath | Shortness of breath at presentation (as recorded) | 1= yes  0= no |
| severe_respiratory_distress | Severe respiratory distress at admission | 1= yes  0= no |
| flu_diag | Method of diagnosis of swine flu whether clinical or laboratory diagnosis (this includes RT-PCR, Direct Fluorescent Antibody test (DFA), Viral Culture, Rapid Antigen test ) | 1= clinical  2= laboratory diagnosis (not otherwise specified) or confirmed using various laboratory tests |
| severe_disease | Severity was defined using standard severity measures such as AVPU, GCS, SOFA or CURB-65 scores where available. Alternatively, the following proxy indicators were used in order of preference: i) severe respiratory distress (tachypnoea, dyspneoa, evidence of alveolar flooding on radiograph, arterial blood gas analysis, cyanosis, excessive respiratory effort) ii) shortness of breath, iii) Symptom score (as defined earlier). | As provided |
| **Treatment** | | |
| antiviral_start_date | Date of start of antiviral treatment | Recorded as dd/mm/yy |
| preadmit_antiviral | Preadmission antiviral (either oseltamivir, zanamivir or peramivir) | 1= yes  0= no |
| oseltamivir_start_date | Date of start of oseltamivir treatment | Recorded as dd/mm/yy |
| preadmit_oseltamivir | Preadmission oseltamivir | 1= yes  0= no |
| zanamivir_start_date | Date of start of zanamivir treatment | Recorded as dd/mm/yy |
| preadmit_zanamivir | Preadmission zanamivir | 1= yes  0= no |
| antibiotic_start_date | Date of start of antibiotic treatment | Recorded as dd/mm/yy |
| hospital_oseltamivir | Oseltamivir given in hospital | 1= yes  0= no |
| hospital_zanamivir | Zanamivir given in hospital | 1= yes  0= no |
| hospital_peramivir | Peramivir given in hospital | 1=yes  0=no |
| hospital_antibiotic | Antibiotics given in hospital | 1= yes  0= no |
| antiviral_anytime | Antiviral administered at any time (whether in the community or in hospital) | 1= yes  0= no |
| early_antiviral1 | Antiviral administered ≤2 days of symptom onset (whether in the community or in hospital) versus no antiviral treatment | 1= early antiviral  0= no antiviral |
| early_antiviral2 | Antiviral administered ≤2 days of symptom onset (whether in the community or in hospital) versus late antiviral treatment | 1= early antiviral  0= late antiviral |
| lt_2days | Antiviral administered >2 days of symptom onset (whether in the community or in hospital) versus no antiviral treatment | 1= late antiviral (>2 days)  0= no treatment |
| oseltamivir_anytime | Oseltamivir administered at any time (whether in the community or in hospital) | 1= yes  0= no |
| early_oseltamivir1 | Oseltamivir administered ≤2 days of symptom onset (whether in the community or in hospital) versus no antiviral treatment | 1= early oseltamivir  0= no antiviral treatment |
| early_oseltamivir2 | Oseltamivir administered ≤2 days of symptom onset (whether in the community or in hospital) versus late oseltamivir (administered >2 days after symptom onset) | 1= early oseltamivir  0= late oseltamivir |
| zanamivir_anytime | Zanamivir administered at any time (whether in the community or in hospital) | 1= yes  0= no |
| early_zanamivir1 | Zanamivir administered ≤2 days of symptom onset (whether in the community or in hospital) versus no antiviral treatment | 1= early zanamivir  0= no antiviral treatment |
| early_zanamivir2 | Zanamivir administered ≤2 days of symptom onset (whether in the community or in hospital) versus late zanamivir (administered >2 days after symptom onset) | 1= early zanamivir  0= late zanamivir |
| peramivir_anytime | Peramivir administered at any time (whether in the community or in hospital); note: peramivir was authorised for emergency use in patients with swine flu during the pandemic in some countries | 1= yes  0= no |
| early_peramivir1 | Peramivir administered ≤2 days of symptom onset (whether in the community or in hospital) versus no antiviral treatment | 1= early peramivir  0= no antiviral treatment |
| early_peramivir2 | Peramivir administered ≤2 days of symptom onset (whether in the community or in hospital) versus late peramivir (administered >2 days after symptom onset) | 1= early peramivir  0= late peramivir |
| non_nai_anytime | Treatment with non-NAI antiviral drugs at any time (amantadine, rimantadine, ribavirin) | 1= yes  0= no |
| hosp_non_nai | In-hospital treatment with non-NAI antiviral drugs (amantadine, rimantadine, ribavirin) | 1= yes  0= no |
| hosp_steroid | New steroids administered in hospital (dexamethasone, hydrocortisone, prednisolone) | 1= yes  0= no |
| **Outcomes** | | |
| discharge_date | Date of discharge from hospital | Recorded as date dd/mm/yy |
| length_of_stay | Length of stay in hospital (whether general ward or ICU) in days | Number of days |
| critical_care | Admission to critical care facilities (ICU) | 1= yes  0= no |
| icu_admit_care | Date of admission to ICU | Recorded as dd/mm/yy |
| icu_discharge_date | Date of ICU discharge | Recorded as dd/mm/yy |
| icu_lengthstay | Length of ICU stay | Recorded as a continuous variables (no. of days) |
| pneumonia | Pneumonia as recorded (whether clinically diagnosed or radiologically diagnosed or discerned from free text chest x-ray or chest CT report findings ) | 1= yes  0= no |
| cxr_pneumonia | Radiologically diagnosed pneumonia (whether as reported or discerned from free text chest x-ray or chest CT report findings) | 1= yes  0= no |
| pneum_diag | Method of diagnosis of pneumonia (clinical or radiological) | 1= radiological diagnosis  0= clinical diagnosis |
| death | Death (as recorded) | 1= yes  0= no |
| dod | Date of death | Recorded as dd/mm/yy |
| death_cause | Primary cause of death | Free text |
| death_cause_code | Cause of death categories | 1= unrelated to flu  2= influenza related  3= sepsis  4= ARDS/ respiratory failure  5= pneumonia  6= multiorgan failure  7= brain death (unspecified)  8= renal failure  9= liver failure  10= shock  11= CVS |
| P | Propensity scores for treatment- yes vs. no | Recorded as a continuous variable between 0 and 1 |
| ps_quintile | Propensity scores categorised into quintiles for each individual study for NAI treatment- yes vs. no | Categorical variable with values from 1 to 5 (1=lowest quintile and 5=highest quintile) |
| p_1 | Propensity scores for treatment- Early treatment (≤2days) vs. no NAI treatment | Recorded as a continuous variable between 0 and 1 |
| ps1_quintile | Propensity scores categorised into quintiles for each individual study for early treatment (≤2days) vs. no treatment | Categorical variable with values from 1 to 5 (1=lowest quintile and 5=highest quintile) |
| p_2 | Propensity scores for treatment- Early treatment (≤2days) vs. Later treatment (>2 days) | Recorded as a continuous variable between 0 and 1 |
| ps2_quintile | Propensity scores categorised into quintiles for each individual study for early treatment (≤2days) vs. later treatment (>2 days) | Categorical variable with values from 1 to 5 (1=lowest quintile and 5=highest quintile) |
| p_lt2 | Propensity scores for treatment- Later treatment (>2days) vs. no NAI treatment | Recorded as a continuous variable between 0 and 1 |
| pslt2_quintile | Propensity scores categorised into quintiles for each individual study for late treatment (>2days) vs. no NAI treatment | Categorical variable with values from 1 to 5 (1=lowest quintile and 5=highest quintile) |

**Figure E1: Date of admission of 29,512 patients hospitalized with A(H1N1)pdm09 infection (by month)**

** Due to scale**,** small numbers of hospitalisations are not visible; there were no hospitalisations in June 2010. Some patients were hospitalised due to other conditions but contracted nosocomial influenza (interval between hospital admission and symptom onset ≥3 days) (n=336; 1.1%).

**Table E4: Comparison of hospitalised patients included in analysis compared with excluded patients**

| **Characteristic** | **All hospitalised patients included in analysis (pneumonia and NAI status known)**  **n (%)** | **All hospitalised patients excluded from in analysis (pneumonia status unknown)**  **n (%)** | **P value** |
| --- | --- | --- | --- |
| Number of patients*, n=29512 | 20634 (69.8) | 8878 (30.1) |  |
| Number of male cases, n= 29502 | 10456 (50.7) | 4114 (46.3) | <0.001 |
| Age: median (IQR) in years , n=29358  Mean (SD) | 25 (10– 44)  28.3 (21.1) | 27 (13– 42)  28.9 (19.6) | 0.0182 |
| Adults (≥16 years), n=19930  Children (<16 years) , n=9367 | 13690 (66.4)  6787 (32.9) | 6240 (70.3)  2580 (29.1) | <0.001 |
| Obese‡ , n= 22782 | 1816 (8.8) | 803 (9.0) | <0.001 |
| Smoking, n=19017 | 1825 (8.8) | 574 (6.5) | <0.001 |
| Pregnant women§ (n=9580) | 1432 (22.5) | 714 (22.2) | <0.001 |
| WHO Regions (n=29512)  African region  Region of the Americas  Eastern Mediterranean Region  European Region  South-East Asia Region  Western Pacific Region | 41 (0.2)  7651 (37.1)  3635 (17.6)  7012 (34.0)  202 (1.0)  2093 (10.1) | 0 (0)  6557 (73.9)  1941 (21.9)  262 (3.0)  8 (0.1)  110 (1.2) | <0.001 |
| A(H1N1)pdm09 diagnosis (n=29512)  Laboratory confirmed  Clinically diagnosed | 20021 (97.0)  613 (3.0) | 5262 (59.3)  3616 (40.7) | <0.001 |
| Comorbidities ¶  Any comorbidity, n=28939  Asthma, n=20524  COPD, n=17085  Other chronic lung disease, n=18117  Heart disease, n=18371  Renal disease, n=20123  Liver disease , n=12207  Cerebrovascular disease, n=9804  Neurological disease, n=13855  Diabetes, n=25023  Immunosuppression, n=25530 | 8980 (43.5)  2398 (11.6)  799 (3.9)  2316 (11.2)  1499 (7.3)  677 (3.3)  248 (1.2)  303 (1.5)  1000 (4.9)  1415 (6.9)  1462 (7.1) | 2062 (23.2)  422 (4.8)  215 (2.4)  185 (2.1)  120 (1.4)  42 (0.5)  43 (0.5)  1 (0.01)  18 (0.2)  672 (7.6)  339 (3.8) | <0.001 |
| H1N1pdm09 vaccination (n=15437) \|\| | 339 (3.4) | 8 (0.2) | <0.001 |
| Time from symptom onset to hospital admission, days, n= 23868  Median (IQR)  Mean(SD) | 2 (1 – 5)  3.31 (5.09) | 2 (1 - 5)  3.14 (5.22) | 0.0169 |
| Antiviral agents used, n=29512  No NAI treatment, n=10453  Any NAI , n=19059  Oral oseltamivir **  Intravenous/inhaled zanamivir **  Intravenous peramivir **  NAI (regimen unknown) **    NAI and Non-NAI **  NAI combination therapy **  Early NAI (≤2 days of symptom onset) **, n=13390  Later NAI (>2 days after symptom onset) **, n=13390 | 5060 (24.5)  15574 (75.5)  15331 (98.4)  313 (2.0)  49 (0.3)  99 (0.6)  94 (0.6)  215 (1.4)  4812 (30.9)  6583 (42.3) | 5393 (60.8)  3485 (39.3)  2207(63.3)  119 (3.4)  0 (0)  1176 (33.7)  0 (0)  17 (0.5)  1275 (36.6)  720 (20.7) | <0.001 |
| Time from symptom onset to antiviral treatment, days, , n=12425  Median (IQR)  Mean (SD) | 3 (2 – 5)  4.07 (4.64) | 2 (1 – 3)  2.59 (4.00) | <0.001 |
| Other in-hospital treatment  Antibiotics, n= 20620  Corticosteroids, n=9987 | 9786 (47.4)  2733 (13.3) | 3418 (38.5)  30 (0.3) | <0.001  <0.001 |
| Hospital length of stay, days, n=22314  Median (IQR)  Mean (SD) | 5 (3 – 11)  9.90 (13.57) | 4 (2 – 7)  5.79 (7.41) | <0.001 |
| Other patient outcomes  Acute respiratory distress syndrome (ARDS), n=6055  Ventilation support, n=15510  Admission to critical care, n=24690  Mortality, n=29487 | 384 (1.9)  3678 (17.8)  5848 (28.3)  1510 (7.3) | 0 (0)  830 (9.4)  1025 (11.6)  1275 (14.4) | <0.001  <0.001  <0.001  <0.001 |

Please add footnotes definitions for no pneumonia, IRP and ‘all pneumonia’ from the main body of the text.

*All percentages have been calculated using these denominators unless otherwise specified.

‡Reported as clinically obese or using WHO definition for obesity (BMI ≥30 kg/m² in adults aged ≥20 years).

§Proportions were calculated as a percentage of pregnant patients among female patients of reproductive age (13–54 years); the broader age range was selected in preference to the WHO definition (15–44 years) after consultation with data contributors to reflect the actual fertility experience of the sample.

¶For definition of comorbidity, see Muthuri et al 2014

||Denominators for pandemic vaccine based on patients admitted after Oct 1, 2009 (when vaccine potentially became available).

**Percentages calculated as a proportion of the sample receiving NAI therapy.

**Table E5: Characteristics of individual studies contributing to the current analysis**

| **Study No.** | **Country** | **Patients source** | **No. of cases with known NAI status** | | | **No. of cases used in primary analysis†** | **Median age, years at hospital admission (Range)** | **Male (%)** | **No. of cases with any comorbidity§ (%)** | **Time to hospital admission, days, median (IQR)** | **No. treated with NAI (%)** | **Time to NAI after symptoms, days, median (IQR)** | **No. of cases with radiographic pneumonia (%)** | **No. of cases admitted to ICU (%)** | **No. of deceased patients (%)** | **Publicationsarising from dataset‡** |
| --- | --- | --- | --- | --- | --- | --- | --- | --- | --- | --- | --- | --- | --- | --- | --- | --- |
| 2 | Bangladesh | Hospital; Surveillance | **141** | | | **70** | 23 (<1 -72) | 85 (60) | 43 (31) | 3 (2 - 5) | 26 (18) | 4 (2 - 6) | 11 (8) | - | 5 (4) | ([1](#_ENREF_1)) |
| 3 | Iran | Hospital;  Single Center | **46** | | | **46** | 32 (15 - 66) | 26 (57) | 19 (41) | 5 (3 - 7) | 46 (100) | 5 (3 - 7) | 9 (20) | 20 (43) | 7 (15) | ([2](#_ENREF_2)) |
| 4 | Spain | ICU;  Multi-center | **1078** | | | **1056** | 47 (1 - 86) | 663 (62) | 571 (53) | 4 (2 - 6) | 1063 (99) | 5 (3 - 7) | 915 (85) | 1078 (100) | 265 (25) | ([3](#_ENREF_3), [4](#_ENREF_4)) |
| 5 | Mexico | Outpatients, Hospitalised; Multi-center | **266** | | | **266** | 39 (<1 - 85) | 161 (61) | 63 (24) | 0 (0 -0) | 266 (100) | 5 ( 0 - 8) | 266 (100) | 76 (29) | 28 (11) | ([5](#_ENREF_5)) |
| 7 | Netherlands | ICU;  Multi-center | **14** | | | **14** | 13 (<1 - 16) | 9 (64) | 8 (57) | - | 14 (100) | 3 (2 - 11) | 13 (93) | 14 (100) | 0 (0) | ([6](#_ENREF_6)) |
| 8 | Hong Kong, China | ICU; Single Center | **17** | | | **17** | 54 (19 - 65) | 11 (65) | 6 (35) | 4 (3 - 7) | 16 (94) | * | 16 (94) | 17 (100) | 1 (6) | - |
| 9 | Lithuania | Hospital;  Multi-center | **121** | | | **94** | 31 (18 - 83) | 52 (43) | 42 (35) | 2 (1 - 3) | 70 (58) | 3 (2 - 6) | 47 (39) | 9 (7) | 6 (5) | ([7](#_ENREF_7)) |
| 10 | Switzerland | Outpatients, Hospitalised; Single Center | **14** | | | **14** | 43 (30 - 82) | 10 (71) | 9 (64) | 5 (2 - 6) | 14 (100) | 7 (3 - 9) | 14 (100) | 6 (43) | 0 (0) | ([8](#_ENREF_8)) |
| 11 | Germany | Hospital; Single Center | **154** | | | **87** | 1 (<1 - 18) | 85 (55) | 98 (64) | 2 (1 - 4) | 42 (27) | - | 51 (33) | 11 (7) | 1 (1) | - |
| 12 | China | Outpatients, Hospitalised; Single Center | **50** | | | **50** | 43 (14 - 75) | 31 (62) | 32 (64) | 5 (4 - 7) | 50 (100) | 5 (4 - 7) | 50 (100) | 34 (68) | 9 (18) | - |
| 13 | China | Hospital;  Multi-center | **155** | | | **151** | 39 (15 - 93) | 90 (58) | 62 (40) | 5 (3 - 7) | 132 (85) | * | 129 (83) | 74 (48) | 27 (17) | ([9](#_ENREF_9)) |
| 14 | Turkey | ICU;  Single Center | **20** | | | **20** | 36 (15 - 72) | 10 (50) | 10 (50) | 5 (3 - 6) | 20 (100) | 5 (4 - 6) | 20 (100) | 20 (100) | 9 (45) | ([10](#_ENREF_10)) |
| 15 | Argentina | Outpatients, Hospitalised; Single Center | **23** | | | **22** | 38 (16 - 82) | 14 (61) | 9 (39) | 3 (2 - 4) | 23 (100) | 3 (2 - 4) | 16 (70) | 7 (30) | 5 (22) | ([11](#_ENREF_11)) |
| 16 | Spain | Outpatients, Hospitalised; Multi-center | **48** | | | **48** | 46 (18 - 84) | 28 (58) | 26 (54) | 5 (3 - 7) | 48 (100) | 6 (3 - 7) | 48 (100) | 11 (23) | 2 (4) | ([12](#_ENREF_12)) |
| 17 | Iran | Outpatients, Hospitalised; Single Center | **143** | | | **76** | 34 (14 - 86) | 66 (46) | 66 (46) | 4 (2 - 6) | 143 (100) | 4 (2 - 6) | 76 (53) | 52 (36) | 17 (12) | ([13](#_ENREF_13)) |
| 18 | Canada | Outpatients, Hospitalised; Single Center | **32** | | | **24** | 5 (<1 - 18) | 20 (63) | 20 (63) | 3 (1 - 5) | 27 (84) | - | 15 (47) | 7 (22) | 0 (0) | ([14](#_ENREF_14)) |
| 19 | South Africa | Hospital;  Single Center | **41** | | | **29** | 27 (<1 - 70) | 4 (10) | 26 (63) | 1 (0 - 4) | 41 (100) | 2 (0 - 4) | 28 (68) | 25 (61) | 14 (34) | ([15](#_ENREF_15)) |
| 20 | Argentina | Hospital;  Multi-center | **112** | | | **75** | 27 (<1 - 79) | 32 (29) | 39 (35) | 2 (1 - 4) | 109 (97) | 3 (1 - 5) | 70 (63) | 43 (38) | 23 (21) | - |
| 21 | Poland | Hospital;  Single Center | **24** | | | **12** | 6 (<1 - 17) | 8 (33) | 8 (33) | - | 24 (100) | 2 (1 - 4) | 6 (25) | 1 (4) | 0 (0) | ([16](#_ENREF_16)) |
| 22 | Jordan | Hospital;  Single Center | **45** | | | **23** | 7 (1 - 19) | 27 (60) | 45 (100) | 5 (4 - 6) | 45 (100) | 1 (1 - 1) | 11 (24) | 6 (13) | 0 (0) | ([17](#_ENREF_17)) |
| 23 | Israel | Outpatients, Hospitalised; Single Center | **37** | | | **28** | 6 (1 - 17) | 19 (51) | 23 (62) | - | 25 (68) | - | 9 (24) | 2 (5) | 0 (0) | ([18](#_ENREF_18)) |
| 24 | Australia | Hospital;  Single Center | **95** | | | **80** | 28 (<1 - 76) | 35 (37) | 62 (65) | - | 84 (88) | - | 25 (26) | 13 (14) | 2 (3) | ([19](#_ENREF_19)) |
| 25 | Turkey | Hospital;  Single Center | **204** | | | **99** | 31 (16 - 86) | 98 (48) | 98 (48) | 3 (2 - 4) | 196 (96) | - | 99 (49) | 19 (9) | 4 (2) | ([20](#_ENREF_20)) |
| 26 | Morocco | Outpatients, Hospitalised; Single Center | **16** | | | **2** | 29 (1 - 42) | 6 (38) | 12 (75) | - | 16 (100) | - | 2 (13) | 2 (13) | 0 (0) | ([21](#_ENREF_21)) |
| 27 | France | Hospital;  Single Center | **1220** | | | **-** | 38 (<1 - 104) | 638 (52) | 616 (50) | 2 (1 - 4) | 1101 (90) | 2 (1 - 5) | - | 881 (72) | 175 (14) | ([22](#_ENREF_22), [23](#_ENREF_23)) |
| 28 | Spain | Hospital;  Multi-center | **698** | | | **656** | 41 (16 - 97) | 366 (52) | 346 (50) | 3 (2 - 5) | 666 (95) | 3 (2 - 6) | 348 (50) | 119 (17) | 37 (5) | ([24](#_ENREF_24), [25](#_ENREF_25)) |
| 29 | Singapore | Outpatients, Hospitalised; Single Center | **339** | | | **143** | 19 (<1 - 93) | 175 (52) | 161 (47) | 2 (1- 3) | 284 (84) | 3 (2 - 5) | 24 (7) | 22 (6) | 3 (1) | ([26](#_ENREF_26)) |
| 30 | Canada | Hospital;  Multi-center | **299** | | | **-** | 6 (<1 - 19) | 176 (59) | 196 (66) | 2 (1 - 5) | 270 (90) | 3 (1 - 5) | - | 131 (44) | 7 (2) | ([27](#_ENREF_27), [28](#_ENREF_28)) |
| 31 | Australia | Hospital;  Single Center | **105** | | | **105** | 42 (15 - 79) | 49 (47) | 71 (68) | 3 (2 - 5) | 89 (85) | 3 (2 - 5) | 49 (47) | 27 (26) | 3 (3) | ([29](#_ENREF_29)) |
| 32 | Argentina | Hospital;  Single Center | **197** | | | **174** | 36 (<1 - 89) | 94 (48) | 117 (59) | 3 (1 - 7) | 197 (100) | 5 (2 - 7) | 147 (75) | 73 (37) | 49 (25) | - |
| 33 | Argentina | ICU, Hospitalised, Outpatients; Single Center | **194** | | | **144** | 33 (<1 - 84) | 97 (50) | 36 (19) | 4 (2 - 6) | 116 (60) | - | 144 (74) | 29 (15) | 27 (14) | - |
| 34 | Spain | Hospital;  Single Center | **91** | | | **84** | 42 (13 - 79) | 37 (41) | 63 (69) | 2 (0 - 3) | 83 (91) | 2 (0 - 4) | 42 (46) | 30 (33) | 7 (8) | ([30](#_ENREF_30)) |
| 35 | Argentina | Outpatients, Hospitalised; Single Center | **68** | | | **38** | 34 (<1 - 80) | 36 (53) | 16 (24) | - | 49 (72) | - | 23 (34) | 16 (24) | 5 (7) | ([31](#_ENREF_31)) |
| 37 | Italy | Hospital;  Single Center | **81** | | | **81** | 32 (1 - 81) | 45 (56) | 43 (53) | - | 69 (85) | 2 (1 - 3) | 51 (63) | 9 (11) | 2 (2) | ([32](#_ENREF_32)) |
| 38 | Turkey | Hospital;  Single Center | **15** | | | **15** | 2 (<1 - 8) | 7 (47) | 1 (7) | 0 (0 - 1) | 15 (100) | 0 (0 - 1) | 3 (20) | 4 (27) | 1 (7) | ([33](#_ENREF_33)) |
| 39 | Israel | Hospital;  Multi-center | **504** | | | **482** | 43 (16 - 93) | 240 (48) | 305 (61) | 3 (1 - 4) | 450 (89) | 3 (2 - 5) | 187 (37) | 34 (7) | 18 (4) | ([34](#_ENREF_34)) |
| 40 | Brazil | Hospital;  Single Center | **21** | | | **20** | 23 (15 - 32) | 0 (0) | 1 (5) | 1 (0 - 2) | 21 (100) | 1 (0 - 2) | 6 (29) | 6 (29) | 0 (0) | ([35](#_ENREF_35)) |
| 41 | France | Outpatients, Hospitalised; Single Center | **84** | | | **-** | 24 (14 - 42) | 0 (0) | 26 (31) | 2 (1 - 2) | 73 (87) | 2 (1 - 2) | - | 1 (1) | 0 (0) | ([36](#_ENREF_36)) |
| 44 | Brazil | Hospital;  Multi-center | **163** | | | **110** | 32 (<1 - 73) | 81 (50) | - | 5 (3 - 7) | 93 (57) | 5 (3 - 7) | 102 (93) | 92 (56) | 49 (30) | ([37](#_ENREF_37)) |
| 45 | Finland | ICU;  Multi-center | **132** | | | **131** | 49 (<1 - 88) | 85 (64) | 96 (73) | 3 (1 - 6) | 126 (95) | 4 (2 - 6) | 103 (78) | 132 (100) | 10 (8) | ([38](#_ENREF_38)) |
| 47 | USA | Hospital;  Single Center | **305** | | | **229** | 6 (<1 - 22) | 186 (61) | 217 (71) | 3 (1 - 5) | 269 (88) | 3 (1 - 5) | 84 (28) | 79 (26) | 8 (3) | ([39](#_ENREF_39)) |
| 48 | Turkey | Hospital; Surveillance | **114** | | | **114** | 6 (<1 - 16) | 68 (60) | 67 (59) | 2 (2 - 4) | 114 (100) | 2 (2 - 4) | 101 (89) | 12 (11) | 0 (0) | ([40](#_ENREF_40)) |
| 49 | Canada | Hospital;  Single Center | **81** | | | **71** | 1 (<1 - 16) | 42 (52) | 38 (47) | 4 (2 - 7) | 54 (67) | 5 (4 - 7) | 55 (68) | 12 (15) | 1 (1) | ([41](#_ENREF_41)) |
| 50 | Denmark | ICU; Surveillance | **51** | | | **41** | 47 (3 - 80) | 30 (59) | 37 (73) | 3 (1 - 6) | 47 (92) | 5 (2 - 8) | 41 (80) | 51 (100) | 18 (35) | ([42](#_ENREF_42)) |
| 51 | Saudi Arabia | Hospital;  Single Center | **127** | | | **126** | 27 (11 - 79) | 100 (79) | 67 (53) | 3 (1 - 4) | 127 (100) | 3 (1 - 4) | 43 (34) | 15 (12) | 4 (3) | ([43](#_ENREF_43)) |
| 52 | Brazil | Outpatients, Hospitalised; Surveillance | **3158** | | | **-** | 26 (<1 - 94) | 1292 (41) | 1339 (42) | - | 557 (18) | - | - | - | 171 (5) | - |
| 53 | Slovenia | Outpatients, Hospitalised; Single Center | **50** | | | **20** | 30 (1 - 87) | 36 (72) | 20 (40) | 3 (1 - 5) | 33 (66) | 4 (2 -6) | 17 (34) | 4 (8) | 3 (6) | - |
| 54 | Japan | Hospital; Single Center | **104** | | | **62** | 8 (<1 - 72) | 54 (52) | 27 (26) | 2 (1 - 3) | 104 (100) | 2 (1 - 3) | 62 (60) | 5 (5) | 1 (1) | ([44](#_ENREF_44)) |
| 55 | India | Hospital;  Single Center | **61** | | | **61** | 22 (<1 - 60) | 35 (57) | 16 (26) | 5 (3 - 7) | 61 (100) | 5 (3 - 7) | 34 (56) | 3 (5) | 9 (15) | - |
| 57 | China | Hospital;  Single Center | **72** | | | **72** | 41 (18 - 66) | 54 (75) | 23 (32) | 8 (6 - 10) | 72 (100) | 7 (4 - 9) | 72 (100) | 35 (49) | 10 (14) | ([45](#_ENREF_45)) |
| 58 | Croatia | Hospital; Single Center | **169** | | | **148** | 29 (<1 - 83) | 95 (56) | 71 (42) | 2 (1 - 4) | 139 (82) | 2 (1 - 5) | 83 (49) | 34 (20) | 4 (2) | ([46](#_ENREF_46)) |
| 59 | UK | Hospital;  Multi-center | **1520** | | | **1014** | 26 (<1 - 95) | 720 (47) | 746 (49) | 2 (1 - 4) | 1154 (76) | 2 (1 - 5) | 254 (17) | 251 (17) | 83 (5) | ([47](#_ENREF_47)) |
| 60 | Poland | Hospital;  Single Center | **13** | | | **11** | 27 (6 - 75) | 4 (31) | 5 (38) | 2 (0 - 2) | 10 (77) | 5 (2 - 9) | 6 (46) | 4 (31) | 3 (23) | - |
| 61 | Slovenia | Hospital;  Single Center | **64** | | | **50** | 39 (17 - 87) | 31 (48) | 37 (58) | 4 (2 - 6) | 62 (97) | 4 (2 - 6) | 28 (44) | 9 (14) | 2 (3) | ([48](#_ENREF_48)) |
| 62 | Serbia | Hospital;  Single Center | **98** | | | **98** | 27 (14 - 88) | 68 (69) | 18 (18) | 3 (1 - 5) | 69 (70) | 3 (1 - 5) | 30 (31) | 6 (6) | 2 (2) | ([49](#_ENREF_49)) |
| 63 | UK | ICU;  Single Center | **24** | | | **24** | 48 (22 - 80) | 13 (54) | 20 (83) | 5 (3 - 8) | 24 (100) | 5 (3 - 9) | 21 (88) | 24 (100) | 7 (29) | ([50](#_ENREF_50)) |
| 64 | Argentina | Hospital;  Multi-center | **250** | | | **-** | 0.8 (<1 - 19) | 130 (52) | 81 (32) | 4 (2 - 7) | 208 (83) | 7 (4 - 10) | - | 46 (18) | 13 (5) | ([51](#_ENREF_51)) |
| 65 | China | Hospital;  Multi-center | **367** | | | **305** | 21 (<1 - 84) | 214 (58) | 52 (14) | 2 (1 - 4) | 362 (99) | 3 (1 - 5) | 149 (41) | 57 (16) | 20 (5) | - |
| 66 | Austria | Outpatients, Hospitalised; Multi-center | **342** | | | **273** | 21 (<1 - 75) | 241 (70) | 123 (36) | - | 242 (71) | - | 90 (26) | 48 (14) | 14 (4) | ([52](#_ENREF_52)) |
| 67 | Iran | Hospital;  Multi-center | **484** | | | **13** | 31 (1 - 84) | 201 (42) | 45 (9) | 3 (2 - 6) | 464 (96) | 3 (2 - 7) | 7 (1) | 44 (9) | 6 (1) | ([53](#_ENREF_53)) |
| 68 | Mongolia | Hospital; Surveillance | **202** | | | **71** | 21 (1 - 76) | 105 (52) | 80 (40) | 3 (1 - 5) | 107 (53) | - | 71 (35) | 6 (3) | 0 (0) | - |
| 69 | Germany | Hospital;  Single Center | **92** | | | **23** | 4 (<1 - 18) | 47 (51) | 13 (14) | 2 (1 - 4) | 28 (30) | 2 (1 - 5) | 15 (16) | 6 (7) | 0 (0) | ([54](#_ENREF_54)) |
| 70 | Australia | Hospital;  Multi-centre | **458** | | | **94** | 4 (<1 - 15) | 266 (58) | 208 (45) | 2 (1 - 4) | 245 (53) | 2 (1 - 6) | 94 (21) | 54 (12) | 3 (1) | ([55](#_ENREF_55)) |
| 71 | Saudi Arabia | Outpatients, Hospitalised; Surveillance | **2676** | | | **-** | 17 (<1 - 90) | 1339 (50) | 597 (22) | 2 (1 - 3) | 2569 (96) | 2 (1 - 4) | - | 234 (9) | 25 (1) | - |
| 73 | USA | ICU;  Multi-center | **630** | | | **595** | 47 (13 - 92) | 285 (45) | 367 (58) | 4 (2 - 7) | 586 (93) | 3 (2 -6) | 506 (80) | 630 (100) | 146 (23) | ([56](#_ENREF_56)) |
| 74 | USA | ICU;  Multi-center | **838** | | | **753** | 6 (<1 - 20) | 485 (58) | 558 (67) | 2 (1 - 5) | 758 (90) | 1 (0 - 3) | 537 (64) | 838 (100) | 74 (9) | ([57](#_ENREF_57)) |
| 75 | UK | Hospital;  Single Center | **34** | | | **28** | 4 (<1 - 15) | 17 (50) | 16 (47) | 3 (1 - 5) | 27 (79) | 4 (1 - 6) | 23 (68) | 14 (41) | 9 (26) | ([58](#_ENREF_58)) |
| 76 | Canada | Hospital;  Single Center | **1014** | | | **343** | 29 (<1 - 92) | 523 (52) | 541 (53) | 3 (1 - 5) | 746 (74) | * | 343 (34) | 167 (16) | 55 (5) | - |
| 77 | Norway | Hospital;  Single Center | **129** | | | **102** | 47 (15 - 93) | 59 (46) | 60 (47) | 3 (1 - 7) | 94 (73) | 3 (1 - 6) | 37 (29) | 7 (5) | 1 (1) | - |
| **SUMMARY OF AVAILABLE DATA** | | | | | | | | | | | | | | | | |
| **ALL STUDIES (with known NAI status) IN THE POOLED DATASET** | | | | | | | | | | | | | | | | |
| TOTAL (69 studies) | | | | **20634** | | **9327** | 25 (<1 -104) | 10456(51) | 8980 (44) | 2 (1 - 5) | 15574 (75) | 3 (2 - 5) | 5978 (29) | 5848 (28) | 1510 (7) |  |
| Patients with known NAI and Pneumonia status , n=69 studies | | | | | **17019** | **9327** | 25 (<1 -104) | 8950 (53) | 7532 (44) | 2 (1 - 5) | 14642 (86) | 3 (2 - 5) | 5978 (35) | 5671 (33) | 1300 (8) |  |
| Patients with known NAI and unknown pneumonia status , n=22 studies | | | | | **3615** | **0** | 25 (<1 - 94) | 1506 (42) | 1448 (40) | 2 (1 – 5) | 932 (26) | 3 (1 - 5) | - | 177 (5) | 210 (6) |  |
| **STUDIES (with known NAI and Pneumonia status) INCLUDED IN PRIMARY ANALYSIS** | | | | | | | | | | | | | | | | |
| TOTAL (63 studies) | | | | | | **9327** | 32 (<1 – 97) | 5145 (55) | 4816 (52) | 3 (1 – 5) | 8205 (88) | 4 (2 - 6) | 5978 (64) | 4099 (44) | 992 (11) |  |
| Datasets included in primary analysis (Table 2), n=49 | | | | | | **7975** | 32 (<1 – 97) | 4398 (55) | 4237 (53) | 3 (2 – 5) | 7051 (88) | 3 (2 - 6) | 4626 (58) | 3693 (46) | 851 (11) |  |
| Datasets solely based on ICU patients included in primary analysis (Table 2), n= 7 | | | | | | **2,590** | 37 (<1 - 92) | 1476 (57) | 1533 (59) | 3 (2 - 6) | 2460 (95) | 5 (3 - 7) | 2111 (82) | 2590 (100) | 483 (19) |  |
| Datasets solely based on radiographic pneumonia cases (excluded from primary analysis – Table 2), n= 14 | | | | | | **1352** | 35 (<1 - 90) | 747 (55) | 579 (43) | 3 (0 – 6) | 1154 (85) | 5 (2 – 7) | 1352 (100) | 406 (30) | 142 (11) |  |
| **Lab and clinically confirmed cases with chest radiographic information and Early (≤2 days) versus Later (>2 days) or No NAI treatment data** | | | | | | | | | | | | | | | | |
| Early vs No NAI treatment (43 studies) | | | | | | **2605** | 29 (<1 -93) | 1468 (56) | 1393 (53) | 2 (1 - 2) | 1835 (70) | 1 (1 - 2) | 1091 (42) | 781 (30) | 176 (7) |  |
| Early vs Late NAI treatment (43 studies) | | | | | | **5058** | 37 (<1 -97) | 2803 (55) | 2705 (53) | 3 (2 – 5) | 5058 (100) | 3 (2 - 6) | 3003 (59) | 2098 (41) | 535 (11) |  |
| Later vs No NAI treatment (43 studies) | | | | | | **3992** | 37 (<1 -97) | 2195 (55) | 1953 (49) | 4 (3 - 6) | 3221 (81) | 5 (4 - 7) | 2536 (64) | 1747 (44) | 494 (12) |  |

ED, Emergency department; IQR, interquartile range (25^th^ and 95^th^ percentile)

†Include hospitalised patients with complete information on NAI treatment and pneumonia status; §comorbidity as a defined in Muthuri et al 2014; ‡ publications emerging from these datasets whether referring specifically to mortality or not; * data supplied as binary variable (Early (≤2 days) versus Later (>2 days) NAI treatment)

**‡Publications arising from each dataset**

1. Azziz-Baumgartner E, Rahman M, Al Mamun A, Haider MS, Zaman RU, Karmakar PC, Nasreen S, Muneer SM-E, Homaira N, Goswami DR. Early Detection of Pandemic (H1N1) 2009, Bangladesh. *Emerging infectious diseases* 2012; 18: 146.

2. Tabarsi P, Moradi A, Marjani M, Baghaei P, Hashemian S, Nadji S, Fakharian A, Mansouri D, Masjedi M, Velayati A. Factors associated with death or intensive care unit admission due to pandemic 2009 influenza A (H1N1) infection. *Annals of Thoracic Medicine* 2011; 6 (2): 91-95.

3. Rodríguez A, Díaz E, Martín-Loeches I, Sandiumenge A, Canadell L, Díaz JJ, Figueira JC, Marques A, Álvarez-Lerma F, Vallés J. Impact of early oseltamivir treatment on outcome in critically ill patients with 2009 pandemic influenza A. *Journal of antimicrobial chemotherapy* 2011; 66: 1140-1149.

4. Rodriguez A, Martin-Loeches I, Bonastre J, Olaechea P, Alvarez-Lerma F, Zaragoza R, Guerrero J, Blanco J, Gordo F, Pozo F, Lorente J, Carratala J, Cordero M, Rello J, Esteban A, Leon C. First influenza season after the 2009 pandemic influenza: Report of the first 300 ICU admissions in Spain *Medicina Intensiva* 2011; 35 208-216.

5. Higuera Iglesias AL, Kudo K, Manabe T, Corcho Berdugo AE, Corrales Baeza A, Alfaro Ramos L, Guevara Gutierrez R, Manjarrez Zavala ME, Takasaki J, Izumi S, Bautista E, Perez Padilla JR. Reducing occurrence and severity of pneumonia due to pandemic H1N1 2009 by early oseltamivir administration: a retrospective study in Mexico. *PloS one* 2011; 6: e21838.

6. van Zwol A, Witteveen R, Markhorst D, Geukers VG. Clinical features of a Dutch cohort of critically ill children due to the 2009 new influenza A H1N1 pandemic. *Clinical pediatrics* 2011; 50: 69-72.

7. Mickienė A, Daniusevičiūtė L, Vanagaitė N, Vėlyvytė D, Blauzdžiūnienė O, Nadišauskienė R, Macas A, Sakalauskas R, Pilvinis V, Nedzelskienė I. Hospitalized adult patients with 2009 pandemic influenza A (H1N1) in Kaunas, Lithuania. *Medicina (Kaunas, Lithuania)* 2011; 47: 11- 18.

8. Bertisch B, Vernazza P, Boggian K. Patients with influenza A/H1N1v-associated pneumonia: the perspective of a tertiary care hospital in Switzerland. *Swiss Med Wkly* 2010; 140: w13069.

9. Cao B, Li X-W, Mao Y, Wang J, Lu H-Z, Chen Y-S, Liang Z-A, Liang L, Zhang S-J, Zhang B, Gu L, Lu L-H, Wang D-Y, Wang C, National Influenza APCIGoC. Clinical features of the initial cases of 2009 pandemic influenza A (H1N1) virus infection in China. *New England Journal of Medicine* 2009; 361: 2507-2517.

10. Sertogullarindan B, Ozbay B, Gunini H, Sunnetcioglu A, Arisoy A, Bilgin HM, Mermit Cilingir B, Duran M, Yildiz H, Ekin S, Baran AI. Clinical and prognostic features of patients with pandemic 2009 influenza A(H1N1) virus in the intensive care unit. *African Health Sciences* 2011; 11: 163-170.

11. Bantar C, Oliva ME, Ré HA, Sandillú M, Franco D, Izaguirre M, Carmagnac A, Vesco E, Grieve CG, Duarte J. Severe acute respiratory disease in the setting of an epidemic of swine-origin type A H1N1 influenza at a reference hospital in Entre Ríos, Argentina. *Clinical infectious diseases* 2009; 49: 1458-1460.

12. Riquelme R, Torres A, Rioseco ML, Ewig S, Cillóniz C, Riquelme M, Inzunza C, Polverino E, Gomez Y, Marcos M. Influenza pneumonia: a comparison between seasonal influenza virus and the H1N1 pandemic. *European Respiratory Journal* 2011; 38: 106-111.

13. Dashti-Khavidaki S, Khalili H, Gholamalipour F, Soudbakhsh A, Talasaz AH, Hajabdolbaghi M, Rasoolinejad M, Azad TM, Fathi M, Talebian MT. Approach to Pandemic 2009 influenza: first report from a main referral hospital for Pandemic H1N1 influenza care in Iran. *The Journal of Infection in Developing Countries* 2010; 4: 629-635.

14. Morris SK, Parkin P, Science M, Subbarao P, Yau Y, O'Riordan S, Barton M, Allen UD, Tran D. A retrospective cross-sectional study of risk factors and clinical spectrum of children admitted to hospital with pandemic H1N1 influenza as compared to influenza A. *BMJ Open* 2012; 2.

15. Langenegger E, Coetzee A, Jacobs S, le Roux A, Theron G. Severe acute respiratory infection with influenza A (H1N1) during pregnancy. *Samj, S* 2009; Suid-Afrikaanse Tydskrif Vir Geneeskunde. 99: 713-714.

16. Radzikowski A, Dembinski L, Talarek E, Smalisz-Skrzypczyk K, Jackowska T, Marczynska M. Pandemic A (H1N1) influenza in hospitalized children in Warsaw, Poland. *Pediatric Infectious Disease Journal* 2011; 30 (1): 90.

17. Amayiri N, Madanat F. Retrospective analysis of pediatric cancer patients diagnosed with the pandemic H1N1 influenza infection. *Pediatric Blood & Cancer* 2011; 56: 86-89.

18. Dubnov‐Raz G, Somech R, Warschawski Y, Eisenberg G, Bujanover Y. Clinical characteristics of children with 2009 pandemic H1N1 influenza virus infections. *Pediatrics International* 2011; 53: 426-430.

19. Keijzers GB, Vossen CNK-L, Zhang P, MacBeth D, Derrington P, Gerrard JG, Doust J. Predicting influenza A and 2009 H1N1 influenza in patients admitted to hospital with acute respiratory illness. *Emergency Medicine Journal* 2011; 28: 500-506.

20. Metan G, Bozkurt I, Agkus C, Coskun R, Alp E, Sungur M, Aygen B, Doganay M. Hospitalized pandemic influenza A (H1N1) patients in a university hospital. *Central European Journal of Medicine* 2011; 6: 83-88.

21. Amine IL, Bajjou T, El Rhaffouli H, Laraqui A, Hilali F, Menouar K, Ennibi K, Boudlal M, Bouaiti EA, Sbai K, Rbai M, Hachim M, Zouhair S. Pandemic influenza A(H1N1) 2009 in Morocco: experience of the Mohammed V Military Teaching Hospital, Rabat, 12 June to 24 December 2009. *Eurosurveillance* 2011; 16: 15-20.

22. Fuhrman C, Bonmarin I, Bitar D, Cardoso T, Duport N, Herida M, Isnard H, Guidet B, Mimoz O, Richard JC, Brun-Buisson C, Brochard L, Mailles A, Paty AC, Saura C, Levy-Bruhl D. Adult intensive-care patients with 2009 pandemic influenza A(H1N1) infection. *Epidemiol Infect* 2011; 139: 1202-1209.

23. Fuhrman C, Bonmarin I, Paty AC, Duport N, Chiron E, Lucas E, Bitar D, Mailles A, Herida M, Vaux S, Levy-Bruhl D. Severe hospitalised 2009 pandemic influenza A(H1N1) cases in France, 1 July-15 November 2009. *Euro Surveillance: Bulletin Europeen sur les Maladies Transmissibles = European Communicable Disease Bulletin* 2010; 15: 14.

24. Viasus D, Pano-Pardo JR, Pachon J, Campins A, Lopez-Medrano F, Villoslada A, Farinas MC, Moreno A, Rodriguez-Bano J, Oteo JA, Martinez-Montauti J, Torre-Cisneros J, Segura F, Gudiol F, Carratala J. Factors associated with severe disease in hospitalized adults with pandemic (H1N1) 2009 in Spain. *Clinical Microbiology and Infection* 2011; 17 (5): 738-746.

25. Viasus D, Pano-Pardo JR, Pachon J, Riera M, Lopez-Medrano F, Payeras A, Farinas MC, Moreno A, Rodriguez-Bano J, Oteo JA, Ortega L, Torre-Cisneros J, Segura F, Carratala J. Timing of Oseltamivir Administration and Outcomes in Hospitalized Adults with Pandemic 2009 Influenza A (H1N1) Virus Infection. *Chest* 2011; 140: 1025-1032.

26. Lee CK, Lee HK, Loh TP, Lai FYL, Tambyah PA, Chiu L, Koay ESC, Tang JW. Comparison of pandemic (H1N1) 2009 and seasonal influenza viral loads, Singapore. *Emerging infectious diseases* 2011; 17: 287-291.

27. Bettinger JA, Sauve LJ, Scheifele DW, Moore D, Vaudry W, Tran D, Halperin SA, Pelletier L. Pandemic influenza in Canadian children: a summary of hospitalized pediatric cases. *Vaccine* 2010; 28: 3180-3184.

28. Tran D, Vaudry W, Moore DL, Bettinger JA, Halperin SA, Scheifele DW, Aziz S. Comparison of children hospitalized with seasonal versus pandemic influenza A, 2004-2009. *Pediatrics* 2012; 130: 397-406.

29. Denholm JT, Gordon CL, Johnson PD, Hewagama SS, Stuart RL, Aboltins C, Jeremiah C, Knox J, Lane GP, Tramontana AR. Hospitalised adult patients with pandemic (H1N1) 2009 influenza in Melbourne, Australia. *Medical Journal of Australia* 2010; 192: 84-86.

30. Giannella M, Alonso M, de Viedma DG, Roa PL, Catalan P, Padilla B, Munoz P, Bouza E. Prolonged viral shedding in pandemic influenza A(H1N1): clinical significance and viral load analysis in hospitalized patients. *Clinical Microbiology and Infection* 2011; 17: 1160-1165.

31. Echavarría M, Querci M, Marcone D, Videla C, Martínez A, Bonvehi P, Carballal G. Pandemic (H1N1) 2009 cases, Buenos Aires, Argentina. *Emerging infectious diseases* 2010; 16: 311-313.

32. Bassetti M, Parisini A, Calzi A, Pallavicini F, Cassola G, Artioli S, Anselmo M, Pagano G, Rezza G, Viscoli C. Risk factors for severe complications of the novel influenza A (H1N1): analysis of patients hospitalized in Italy. *Clinical Microbiology and Infection* 2011; 17: 247-250.

33. Ozkan M, Tuygun N, Erkek N, Aksoy A, Yıldız YT. Neurologic manifestations of novel influenza A (H1N1) virus infection in childhood. *Pediatric neurology* 2011; 45: 72-76.

34. Hiba V, Chowers M, Levi-Vinograd I, Rubinovitch B, Leibovici L, Paul M. Benefit of early treatment with oseltamivir in hospitalized patients with documented 2009 influenza A (H1N1): retrospective cohort study. *Journal of antimicrobial chemotherapy* 2011; 66: 1150-1155.

35. Jimenez MF, El Beitune P, Salcedo MP, Von Ameln AV, Mastalir FP, Braun LD. Outcomes for pregnant women infected with the influenza A (H1N1) virus during the 2009 pandemic in Porto Alegre, Brazil. *Int J Gynaecol Obstet* 2010; 111: 217-219.

36. Gérardin P, El Amrani R, Cyrille B, Gabrièle M, Guillermin P, Boukerrou M, Boumahni B, Randrianaivo H, Winer A, Rouanet J-F. Low clinical burden of 2009 pandemic influenza A (H1N1) infection during pregnancy on the island of La Reunion. *PloS one* 2010; 5: e10896.

37. Yokota RT, Skalinski LM, Igansi CN, de Souza LR, Iser BP, Reis PO, Barros EN, Macário EM, Bercini MA, Ranieri TM. Risk factors for death from pandemic (H1N1) 2009, southern Brazil. *Emerging infectious diseases* 2011; 17: 1467-1471.

38. Linko R, Pettilä V, Ruokonen E, Varpula T, Karlsson S, Tenhunen J, Reinikainen M, Saarinen K, Perttilä J, Parviainen I, Ala-Kokko T, FINNH1N1-STUDY GROUP. Corticosteroid therapy in intensive care unit patients with PCR-confirmed influenza A(H1N1) infection in Finland. *Acta Anaesthesiologica Scandinavica* 2011; 55: 971-979.

39. Bagdure D, Curtis DJ, Dobyns E, Glodé MP, Dominguez SR. Hospitalized children with 2009 pandemic influenza A (H1N1): comparison to seasonal influenza and risk factors for admission to the ICU. *PloS one* 2010; 5: e15173.

40. Torun SH, Somer A, Salman N, Ciblak M, Demirkol D, Kanturvardar M, Badur S, Devecioğlu Ö. Clinical and epidemiological characteristics of pandemic influenza A/(H1N1) in hospitalized pediatric patients at a university hospital, Istanbul, Turkey. *Journal of tropical pediatrics* 2011; 57: 213-216.

41. Fanella ST, Pinto MA, Bridger NA, Bullard JM, Coombs JM, Crockett ME, Olekson KL, Poliquin PG, Van Caeseele PG, Embree JE. Pandemic (H1N1) 2009 influenza in hospitalized children in Manitoba: nosocomial transmission and lessons learned from the first wave. *Infection Control and Hospital Epidemiology* 2011; 32: 435-443.

42. Gubbels S, Perner A, Valentiner-Branth P, Molbak K. National surveillance of pandemic influenza A (H1N1) infection-related admissions to intensive care units during the 2009-10 winter peak in Denmark: two complementary approaches. *Euro Surveill* 2010; 15: pii=19743.

43. Al-Khuwaitir TS, Al-Abdulkarim AS, Abba AA, Yousef AM, El-Din MA, Rahman KT, Ali MA, Mohamed ME, Arnous NE. H1N1 influenza A. Preliminary evaluation in hospitalized patients in a secondary care facility in Saudi Arabia. *Saudi Med J* 2009; 30: 1532-1536.

44. Kudo K, Takasaki J, Manabe T, Uryu H, Yamada R, Kuroda E, Kobayashi N, Matsushita T. Systemic Corticosteroids and Early Administration of Antiviral Agents for Pneumonia with Acute Wheezing due to Influenza A (H1N1) pdm09 in Japan. *PloS one* 2012; 7: e32280.

45. Cui W, Zhao H, Lu X, Wen Y, Zhou Y, Deng B, Wang Y, Wang W, Kang J, Liu P. Factors associated with death in hospitalized pneumonia patients with 2009 H1N1 influenza in Shenyang, China. *BMC Infectious Diseases* 2010; 10: 145.

46. Čeljuska-Tošev E, Kuzman I, Draženović V, Knezović I, Čivljak R. Clinical and epidemiological characteristics of hospitalized patients with pandemic A (H1N1) 2009 influenza. *Infektološki Glasnik* 2010; 30: 149-159.

47. Myles PR, Semple MG, Lim WS, Openshaw PJ, Gadd EM, Read RC, Taylor BL, Brett SJ, McMenamin J, Enstone JE, Armstrong C, Bannister B, Nicholson KG, Nguyen-Van-Tam JS. Predictors of clinical outcome in a national hospitalised cohort across both waves of the influenza A/H1N1 pandemic 2009-2010 in the UK. *Thorax* 2012; 67: 709-717.

48. Pecavar B, Nadrah K, Papst L, Cec V, Kotar T, Maticic M, Meglic-Volkar J, Vidmar L, Beovic B. Clinical characteristics of adult patients with influenza-like illness hospitalized in general ward during Influenza A H1N1 pandemic 2009/2010. *Wien Klin Wochenschr* 2011; 123: 662-667.

49. Mikic D, Nozic D, Kojic M, Popovic S, Hristovic D, Dimitrijevic RR, Curcic P, Milanovic M, Glavatovic R, Kupresanin VB, Veljovic M, Djordjevic D, Kapulica NK, Cekanac R, Stefanovic D. Clinical manifestations, therapy and outcome of pandemic influenza A (H1N1) 2009 in hospitalized patients. *Vojnosanit Pregl* 2011; 68: 248-256.

50. Yeung JH, Bailey M, Perkins GD, Smith FG. Presentation and management of critically ill patients with influenza A (H1N1): a UK perspective. *Crit Care* 2009; 13: 426; author reply 426.

51. Libster R, Bugna J, Coviello S, Hijano DR, Dunaiewsky M, Reynoso N, Cavalieri ML, Guglielmo MC, Areso MS, Gilligan T, Santucho F, Cabral G, Gregorio GL, Moreno R, Lutz MI, Panigasi AL, Saligari L, Caballero MT, Egues Almeida RM, Gutierrez Meyer ME, Neder MD, Davenport MC, Del Valle MP, Santidrian VS, Mosca G, Garcia Dominguez M, Alvarez L, Landa P, Pota A, Bolonati N, Dalamon R, Sanchez Mercol VI, Espinoza M, Peuchot JC, Karolinski A, Bruno M, Borsa A, Ferrero F, Bonina A, Ramonet M, Albano LC, Luedicke N, Alterman E, Savy V, Baumeister E, Chappell JD, Edwards KM, Melendi GA, Polack FP. Pediatric hospitalizations associated with 2009 pandemic influenza A (H1N1) in Argentina. *New England Journal of Medicine* 2010; 362: 45-55.

52. Poeppl W, Hell M, Herkner H, Stoiser B, Fritsche G, Schurz-Bamieh N, Poeppl G, Gattringer R, Jones N, Maass M, Egle A, Burgmann H. Clinical aspects of 2009 pandemic influenza A (H1N1) virus infection in Austria. *Infection* 2011; 39: 341-352.

53. Moghadami M, Kazeroni PA, Honarvar B, Ebrahimi M, Bakhtiari H, Akbarpour MA, Tabatabaee HR, Mirahmadizadeh AR, Rezaianzadeh A, Hasanzadeh J, Zamiri N, Sabayan B, Moattari A, Emami A, Lankarani KB. Influenza a (H1N1) virus pandemic in fars province: A report from Southern Iran, July-December 2009. *Iranian Red Crescent Medical Journal* 2010; 12 (3): 231-238.

54. Boxhammer S, Lepler R, Lenhartz H, Pust B, Hoger PH. Pandemic influenza A/H1N1-2009 in children and adolescents in Hamburg: Symptoms and course of disease in 95 hospitalized pediatric patients. *Monatsschr Kinderheilkd* 2011; 159: 560-564.

55. Khandaker G, Rashid H, Zurynski Y, Richmond PC, Buttery J, Marshall H, Gold M, Walls T, Whitehead B, Elliott EJ, Booy R. Nosocomial vs community-acquired pandemic influenza A (H1N1) 2009: A nested case-control study. *Journal of Hospital Infection* 2012; 82: 94-100.

56. Rice TW, Rubinson L, Uyeki TM, Vaughn FL, John BB, Miller RR, Higgs E, Randolph AG, Smoot BE, Thompson BT. Critical illness from 2009 pandemic influenza A virus and bacterial coinfection in the United States. *Critical Care Medicine* 2012; 40: 1487-1498.

57. Randolph AG, Vaughn F, Sullivan R, Rubinson L, Thompson BT, Yoon G, Smoot E, Rice TW, Loftis LL, Helfaer M, Doctor A, Paden M, Flori H, Babbitt C, Graciano AL, Gedeit R, Sanders RC, Giuliano JS, Zimmerman J, Uyeki TM, Pediatric Acute Lung I, Sepsis Investigator's N, the National Heart L, Blood Institute ACTN. Critically ill children during the 2009-2010 influenza pandemic in the United States. *Pediatrics* 2011; 128: e1450-1458.

58. Herberg JA, Jones KDJ, Paulus S, Gormley S, Muir D, Cooper M, Levin M. Comparison of pandemic and seasonal influenza reveals higher mortality and increased prevalence of shock in children with severe H1N1/09 infection. *Pediatric Infectious Disease Journal* 2011; 30 438-440.

**Table E6: Sensitivity analysis excluding all ICU patients**

**Association between NAI treatment and influenza-related pneumonia**

| **Subgroups** | **Radiologically confirmed pneumonia** | |
| --- | --- | --- |
|  | **Crude OR (95% CI)** | **Adjusted‡ OR (95% CI)** |
| 1. ***Early NAI (≤2 days) vs. No NAI treatment*** | | |
| Lab and clinically confirmed (all ages) , n=2142 | 0.88 (0.69 to 1.14) | **0.73 (0.55 to 0.96)*** |
| Lab confirmed cases (all ages) , n=2002 | 0.88 (0.68 to 1.14) | **0.73 (0.55 to 0.97)*** |
| 1. ***Early NAI (≤2 days) vs. Later (>2 days)*** | | |
| Lab and clinically confirmed (all ages) , n=3779 | **0.33 (0.28 to 0.38)***** | **0.42 (0.35 – 0.49)***** |
| Lab confirmed cases (all ages) , n=3564 | **0.33 (0.28 to 0.39)***** | **0.42 (0.35 – 0.50)***** |
| 1. ***Later (>2 days) vs None*** | | |
| Lab and clinically confirmed (all ages) , n=2917 | **2.44 (1.93 to 3.09)***** | **1.68 (1.30 to 2.16)***** |
| Lab confirmed cases (all ages) , n=2754 | **2.41 (1.88 to 3.08)***** | **1.64 (1.25 to 2.14)***** |
| 1. ***NAI anytime vs. No NAI treatment:*** | | |
| Lab and clinically confirmed (all ages), n=5385 | **1.48 (1.22 to 1.79)***** | **1.27 (1.03 to 1.57)*** |
| Lab confirmed cases (all ages) , n=5040 | **1.45 (1.18 to 1.77)***** | 1.23 (0.97 to 1.53) |

‡adjusted for treatment propensity quintiles, corticosteroid use and antibiotic use

* P < 0.05, ** P < 0.01, *** P < 0.001

**Table E7: Association between NAI treatment and pneumonia (All children)**

| **Subgroups** | **Influenza-related pneumonia (IRP)** | | **Any Pneumonia†*** | |
| --- | --- | --- | --- | --- |
|  | **Crude OR (95% CI)** | **Adjusted‡ OR (95% CI)** | **Crude OR (95% CI)** | **Adjusted‡ OR (95% CI)** |
| 1. ***Early NAI (≤2 days) vs. No NAI treatment*** | | | | |
| All Children (< 16 years);  (n1=670 ; n2=2765) | 1.04 (0.61 – 1.77) | 0.76 (0.42 – 1.36) | 0.89 (0.69 – 1.14) | 0.78 (0.59 – 1.03) |
| Children (< 5 years);  (n1=317 ; n2=1328) | 0.72 (0.33 – 1.59) | 0.54 (0.22 – 1.36) | 0.71 (0.49 – 1.01) | **0.67 (0.45 – 1.00)*** |
| Children (≥5 < 16 years);  *(n1=353 ; n2=1437)* | 1.40 (0.68 – 2.87) | 1.00 (0.46 – 2.20) | 1.16 (0.81 – 1.66) | 0.92 (0.62 – 1.37) |
| 1. ***Early NAI (≤2 days) vs. Later NAI (>2 days)*** | | | | |
| All Children (< 16 years);  (n1=864 ; n2=3295) | **0.43 (0.29 – 0.62)***** | **0.47 (0.32 – 0.71)***** | **0.43 (0.35 – 0.53)***** | **0.53 (0.43 – 0.66)***** |
| Children (< 5 years);  (n1=376 ; n2=1537) | **0.40 (0.22 – 0.71)**** | **0.35 (0.18 – 0.68)**** | **0.39 (0.29 – 0.53)***** | **0.48 (0.35 – 0.66)***** |
| Children (≥5 < 16 years);  *(n1=488 ; n2=1758)* | **0.44 (0.27 – 0.71)**** | **0.53 (0.31 – 0.89)*** | **0.46 (0.35 – 0.60)***** | **0.57 (0.43 – 0.76)***** |
| 1. ***Later (>2 days) vs No NAI treatment:*** | | | | |
| All Children (< 16 years);  (n1=724 ; n2=2632) | **2.26 (1.28 – 3.99)**** | 1.67 (0.89 – 3.16) | **1.99 (1.55 – 2.57)***** | **1.42 (1.08 – 1.87)**** |
| Children (< 5 years);  (n1=369 ; n2=1433) | 1.97 (0.82 – 4.73) | 1.51 (0.57 – 3.98) | **1.83 (1.30 – 2.57)**** | 1.34 (0.93 – 1.94**)** |
| Children (≥5 < 16 years);  *(n1=355 ; n2=1199)* | **2.95 (1.38 – 6.31)**** | 2.02 (0.84 – 4.85) | **2.39 (1.63 – 3.49)***** | **1.55 (1.02 – 2.35)*** |
| 1. ***NAI anytime vs. No NAI treatment:*** | | | | |
| All Children (< 16 years);  (n1=2005 ; n2=6760) | **1.38 (1.00 – 1.90)*** | 1.30 (0.92 – 1.82) | **1.41 (1.18 – 1.69)***** | 1.18 (0.97 – 1.43) |
| Children (< 5 years);  (n1=949 ; n2=3307) | 1.20 (0.77 – 1.85) | 1.08 (0.68 – 1.72) | 1.25 (0.98 – 1.58) | 1.05 (0.81 – 1.36) |
| Children (≥5 < 16 years);  *(n1=1056 ; n2=3453)* | **1.79 (1.10 – 2.91)*** | **1.70 (1.02 – 2.85)*** | **1.74 (1.31 – 2.30)***** | **1.35 (1.00 – 1.82)*** |

† Influenza related pneumonia (IRP) and physician diagnosed pneumonia (PDP)

‡adjusted for treatment propensity quintiles, corticosteroid use and antibiotic use

n1= total number of patients included in IRP analysis; n2= total number of patients included in ‘any pneumonia’ analysis

* P < 0.05, ** P < 0.01, *** P < 0.001

**Table E8: Association between NAI treatment and pneumonia (Critically ill children)**

| **Subgroups** | **Influenza-related pneumonia (IRP)** | | **Any Pneumonia†*** | |
| --- | --- | --- | --- | --- |
|  | **Crude OR (95% CI)** | **Adjusted‡ OR (95% CI)** | **Crude OR (95% CI)** | **Adjusted‡ OR (95% CI)** |
| 1. ***Early NAI (≤2 days) vs. No NAI treatment*** | | | | |
| All Children (< 16 years);  (n1=197 ; n2=447) | 1.51 (0.58 – 3.97) | 1.33 (0.47 – 3.78) | 1.75 (0.99 – 3.12) | 1.44 (0.77 – 2.62) |
| Children (< 5 years);  (n1=96 ; n2=226) | 1.55 (0.33 – 7.38) | 1.23 (0.23 – 6.64) | 1.70 (0.73 – 3.97) | 1.32 (0.53 – 3.25) |
| Children (≥5 < 16 years);  *(n1=101 ; n2=221)* | 1.55 (0.47 – 5.08) | 1.10 (0.29 – 4.18) | 1.78 (0.84 – 3.77) | 1.20 (0.53 – 2.71) |
| 1. ***Early NAI (≤2 days) vs. Later NAI (>2 days)*** | | | | |
| All Children (< 16 years);  (n1=251 ; n2=655) | **0.46 (0.22 – 0.94)*** | 0.45 (0.20 – 1.01) | **0.61 (0.42 – 0.89)*** | 0.71 (0.47 – 1.05) |
| Children (<5 years);  (n1=107 ; n2=320) | **0.23 (0.06 – 0.94)*** | 0.22 (0.04 – 1.21) | **0.46 (0.25 – 0.83)*** | **0.53 (0.28 – 0.99)*** |
| Children (≥5 < 16 years);  *(n1=144 ; n2=335)* | 0.58 (0.25 – 1.34) | 0.64 (0.25 – 1.63) | 0.68 (0.41 – 1.11) | 0.79 (0.47 – 1.33) |
| 1. ***Later (>2 days) vs No NAI treatment:*** | | | | |
| All Children (< 16 years);  (n1=236 ; n2=518) | **5.84 (1.50 – 22.75)*** | **4.25 (1.07 – 16.88)*** | **3.50 (1.90 – 6.46)***** | **2.63 (1.39 – 4.96)**** |
| Children (< 5 years);  (n1=113 ; n2=268) | 8.26 (0.61 – 112.12) | NC | **3.51 (1.51 – 8.15)**** | **3.05 (1.26 – 7.39)*** |
| Children (≥5 < 16 years);  *(n1=123 ; n2=250)* | **4.50 (1.07 – 18.96)*** | 2.40 (0.54 – 10.75) | **3.27 (1.41 – 7.55)**** | 1.95 (0.82 – 4.68) |
| 1. ***NAI anytime vs. No NAI treatment:*** | | | | |
| All Children (< 16 years);  (n1=970 ; n2=1579) | 1.45 (0.89 – 2.38) | 1.39 (0.85 – 2.29) | **1.75 (1.22 – 2.53)**** | **1.59 (1.10 – 2.30)*** |
| Children (< 5 years);  (n1=425 ; n2=743) | 1.42 (0.72 – 2.81) | 1.41 (0.71 – 2.81) | **1.72 (1.05 – 2.82)*** | 1.67 (0.99 – 2.69) |
| Children (≥5 < 16 years);  *(n1=545 ; n2=836)* | 1.53 (0.75 – 3.14) | 1.48 (0.71 – 3.08) | **1.75 (1.03 – 2.99)*** | 1.46 (0.84 – 2.53) |

† Influenza related pneumonia (IRP) and physician diagnosed pneumonia (PDP)

‡adjusted for treatment propensity quintiles, corticosteroid use and antibiotic use

n1= total number of patients included in IRP analysis; n2= total number of patients included in ‘any pneumonia’ analysis; NC – not computed

* P < 0.05, ** P < 0.01, *** P < 0.001

**Table E9: Stratified analysis based on steroid use:**

| ***Early NAI (≤2 days) vs. No NAI treatment*** | **Crude OR (95% CI) for IRP** | **Adjusted OR † (95% CI) for IRP** |
| --- | --- | --- |
| Lab confirmed and clinically diagnosed H1N1 (all ages) (number included in analysis=559) | 0.82 (0.45 to 1.49) | 0.79 (0.42 to 1.49) |
| Lab confirmed H1N1 (all ages)  (number included in analysis=552) | 0.83 (0.45 to 1.51) | 0.80 (0.42 to 1.50) |

**Patients who were NOT administered in-hospital steroids**)**:**

| ***Early NAI (≤2 days) vs. No NAI treatment*** | **Crude OR (95% CI)** | **Adjusted OR † (95% CI)** |
| --- | --- | --- |
| Lab confirmed and clinically diagnosed H1N1 (all ages) (number included in analysis=1,718) | 0.93 (0.70 to 1.24) | 0.78 (0.58 to 1.07) |
| Lab confirmed H1N1 (all ages)  (number included in analysis=1,612) | 0.88 (0.66 to 1.17) | 0.74 (0.54 to 1.02) |

† Adjusted for treatment propensity quintiles and antibiotic use
